# A simple genetic basis of adaptation to a novel thermal environment results in complex metabolic rewiring in *Drosophila*

**DOI:** 10.1186/s13059-018-1503-4

**Published:** 2018-08-20

**Authors:** François Mallard, Viola Nolte, Ray Tobler, Martin Kapun, Christian Schlötterer

**Affiliations:** 1 Institut für Populationsgenetik, Vetmeduni Vienna, Vienna, Austria; 2 Vienna Graduate School of Population Genetics, Vetmeduni Vienna, Vienna, Austria; 3 Present address: Australian Centre for Ancient DNA, School of Biological Sciences, University of Adelaide, Adelaide, South Australia Australia; 4 Present address: Department of Ecology and Evolution, Université de Lausanne, Lausanne, Switzerland

**Keywords:** Experimental evolution, Evolve and resequence, Thermal adaptation, Pool-Seq, RNASeq

## Abstract

**Background:**

Population genetic theory predicts that rapid adaptation is largely driven by complex traits encoded by many loci of small effect. Because large-effect loci are quickly fixed in natural populations, they should not contribute much to rapid adaptation.

**Results:**

To investigate the genetic architecture of thermal adaptation — a highly complex trait — we performed experimental evolution on a natural *Drosophila simulans* population. Transcriptome and respiration measurements reveal extensive metabolic rewiring after only approximately 60 generations in a hot environment. Analysis of genome-wide polymorphisms identifies two interacting selection targets, *Sestrin* and *SNF4Aγ*, pointing to AMPK, a central metabolic switch, as a key factor for thermal adaptation.

**Conclusions:**

Our results demonstrate that large-effect loci segregating at intermediate allele frequencies can allow natural populations to rapidly respond to selection. Because *SNF4Aγ* also exhibits clinal variation in various *Drosophila* species, we suggest that this large-effect polymorphism is maintained by temporal and spatial temperature variation in natural environments.

**Electronic supplementary material:**

The online version of this article (10.1186/s13059-018-1503-4) contains supplementary material, which is available to authorized users.

## Background

One of the major challenges in evolutionary genetics is to unravel the genetic architecture of phenotypic traits and how this affects the potential of natural populations to respond to selective forces. Theory predicts that evolution proceeds mainly through polygenic quantitative traits [1]. Thus, adaptation is expected to involve rather subtle allele frequency changes at many small-effect loci [2–4]. Classic association studies as well as recent whole genome association studies confirmed that variation at quantitative traits is due to a large number of loci and that large-effect loci are rare [5–7]. Although, association studies are helpful to describe the genetic architecture of phenotypic traits, these genetic variants cannot be directly linked to adaptive responses [8]. In contrast to these theoretical predictions, an increasing number of studies identified a small number of large-effect loci which are driving rapid adaptation (reviewed in [9]). Fluctuating selective pressure across time and space may contribute to the persistence of polymorphism at large-effect loci even over long evolutionary time scales [9–11]. Importantly, these major-effect loci typically encoded rather simple traits, such as melanism [12, 13], insecticide resistance [14], or lactose tolerance in humans [15]. One noticeable exception is the evolution of song-less crickets, which occurred on two islands, but involved different major-effect loci [16]. It remains unclear to what extent rapid adaptation by large-effect loci is an exception of simple traits, which are maintained in the population by fluctuating selection pressures.

In the light of global warming, it is of key interest to understand how novel thermal environments can drive genetic adaptation and the true nature of the associated phenotypic changes [17, 18]. Temperature is a major abiotic factor known to affect a broad range of phenotypes and provides a good study system to investigate the genetic architecture of phenotypic evolution, in particular of quantitative traits. Most insight into thermal adaptation comes from contrasting natural populations that have evolved in different thermal habitats [19–24]. Apart from complications intrinsic to natural populations, such as confounding signals of demography [25] and the complexity of natural environments, the underlying evolutionary time scales are too long to be informative about rapid adaptation required to counter the current rate of climate change [8]. The well-documented clinal variation and seasonal response of many genetic polymorphisms [21–24] make *Drosophila* an excellent model system to study the impact of large-effect alleles segregating in natural populations on rapid adaptation to novel thermal environments. Shared clinal polymorphisms between two sister species, *D. melanogaster* and *D. simulans*, suggest that genetic variants associated with thermal clines may have been segregating for long evolutionary times. Such alleles contributing to temperature adaptation could be readily selected, either in natural populations or in the laboratory, and mediate a fast adaptation to a new temperature regime.

Here we use experimental evolution in *D. simulans* to investigate the genetic architecture of phenotypic adaptation to a novel thermal environment. Transcriptomic data suggest that the evolved populations underwent a massive metabolic rewiring, which was confirmed by resting metabolism measurements. Whole genomic resequencing after ~ 60 generations of evolution under our hot environment indicated that, despite temperature adaptation being a complex trait, only a small number of selection targets were identified across five replicate populations. Two interacting loci were associated with AMP-activated protein kinase (AMPK), a key metabolic switch driving the phenotypic changes observed in our experiment. We show that these alleles are segregating at intermediate frequency in a European population and show a latitudinal cline in North American populations. These results suggest that experimental evolution identified variants which play a key role in rapid spatial and temporal temperature adaptation in natural populations.

## Results

### Experimental evolution and phenotypic response

**Fig. 1:**
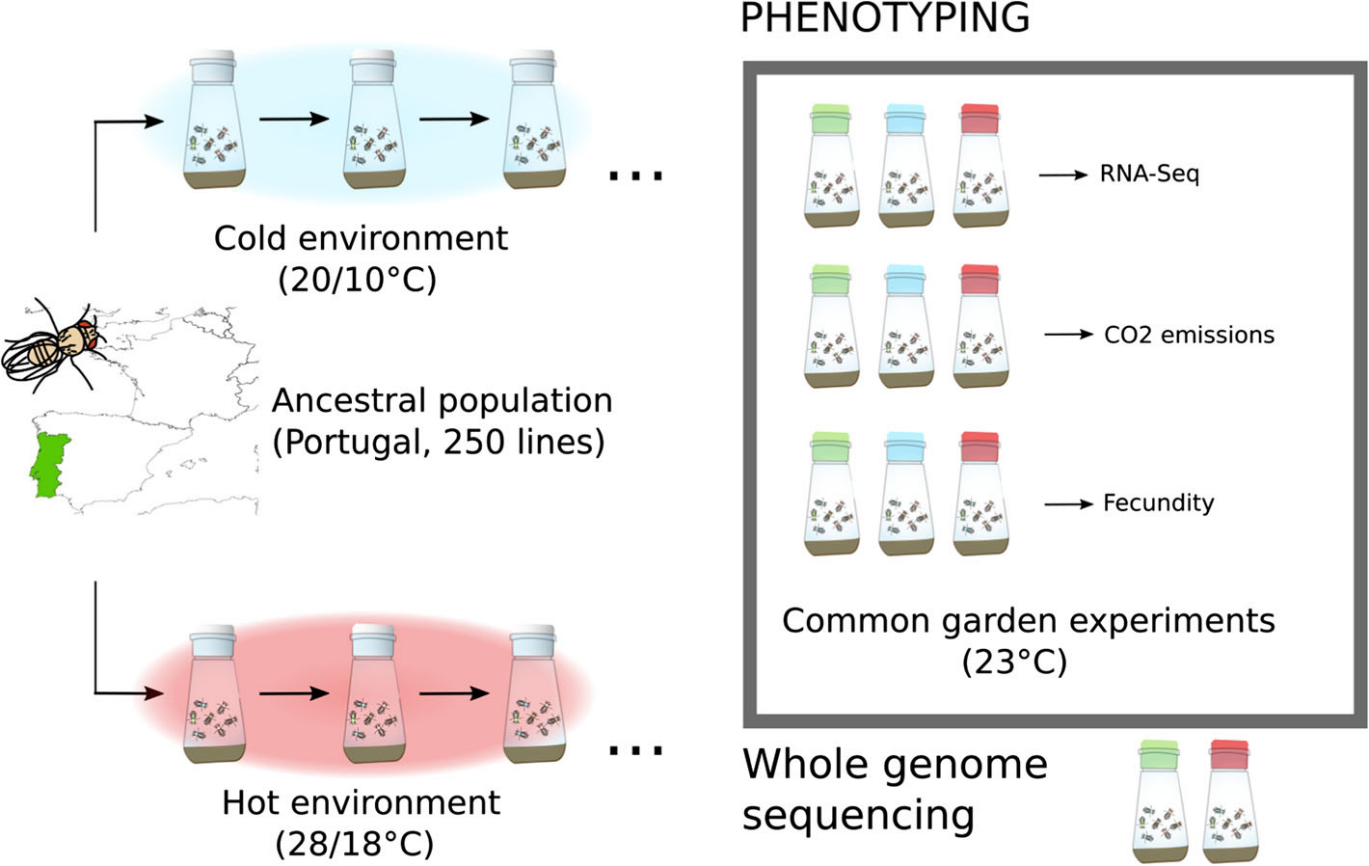
Experimental design: 250 isofemale lines from a natural *Drosophila simulans* population constituted the ancestral populations. The populations were kept at a constant population size (1000 flies) with non-overlapping generations either in a hot environment (*red*) fluctuating between 28 and 18 °C on a 12/12 h cycle or a cold environment (*blue*), fluctuating between 20 and 10 °C (five replicated populations in each environment). We performed multiple phenotypic measurements on the evolved and reconstituted ancestral populations (*green*) in common garden experiments. We profiled the transcriptome of hot- (F64) and cold- (F39) evolved and reconstituted ancestral populations. Resting metabolism (CO_2_ emission) and fecundity were measured later in the experiment (cold-evolved F74–F77; hot-evolved F127–F133). We also sequenced the entire genome of the ancestral and hot-evolved populations (F59)

A natural *D. simulans* population from Póvoa de Varzim, Portugal was selected for ~ 60 generations in a hot environment that fluctuated daily between 18 and 28 °C (Fig. 1). Five independently hot-evolved populations were compared to reconstituted ancestral populations and five populations that evolved in a cold environment fluctuating between 10 and 20 °C. The cold-evolved control populations allowed us to rule out adaptation to culture conditions not specific to the hot environment (i.e., laboratory adaptation). To characterize the adaptive response of the evolved flies to high temperature, we assayed three phenotypes — fecundity, the whole transcriptome, and resting metabolism — in a common garden experiment at 23 °C, the mean temperature of the experimental hot environment.

**Fig. 2:**
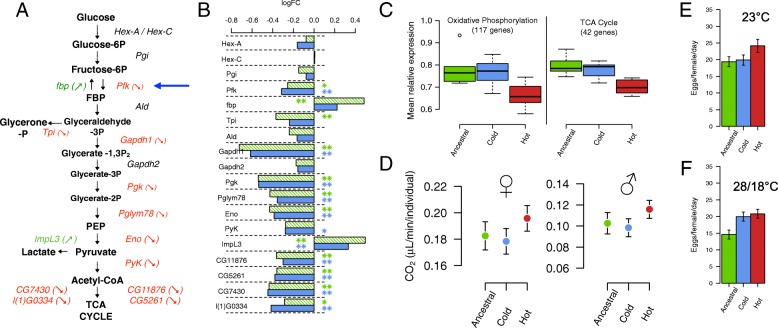
Phenotypic response of hot-evolved flies. **a** Evolution of gene expression in the glycolysis pathway. Enzymes significantly up- (*green*) and down- (*red*) regulated in the hot-evolved populations relative to the ancestral population are shown in *color*. **b** Gene expression changes (log_2_ fold change) in the hot-evolved populations relative to the ancestral (*green*) or cold-evolved (*blue*) populations. (** FDR < 0.05, * FDR < 0.1). **c** Hot-evolved populations (*red*) differ in gene expression from the ancestral (*green*) and cold-evolved (*blue*) populations for genes of the oxidative phosphorylation pathway and TCA cycle. **d** For both sexes, the resting metabolism of hot-evolved flies differs significantly from that of ancestral and cold-evolved populations. Both ancestral and cold-evolved populations are significantly different from the hot-evolved populations when considering both sexes in a single model (*p* = 0.032 and *p* = 0.003, respectively). *Bars* show mean ± 95% confidence intervals as estimated by our linear model (see Methods). **e**, **f** Higher fitness of hot-evolved flies: hot-evolved flies have a higher fecundity than the ancestral (*p* = 0.0006) or cold-evolved populations (*p* = 0.0018) at 23 °C but differ only from the ancestral populations at 28/18 °C (*p* < 0.0001), which is consistent with previous results in *D. melanogaster* (see Additional file 1: Supplementary Methods and Results for a detailed discussion)

Consistent with an adaptive response, the hot-evolved *D. simulans* populations were fitter than the ancestral population in the hot environment. In common garden experiments involving two different hot temperature regimes, the hot-evolved populations were more fecund (total number of eggs laid over successive 5 days) than the ancestral population (*p* = 0.0006 and *p* < 0.0001 at 23 °C and 18/28 °C cycling, respectively, Fig. 2e, f). Similar to previous observations in *D. melanogaster* [26], hot- and cold-evolved populations were only significantly different from each other at 23 °C (*p* = 0.0018, Fig. 2e), but not in the fluctuating temperature regime. These fitness differences suggest that the flies in our experiment adapted to a higher mean temperature as well as rapid temperature fluctuations [26].

We further characterized the molecular phenotype of the hot-evolved and control flies using RNA sequencing (RNA-seq), collecting whole transcriptome data of young adult males (3–5 days old). Out of more than 9000 genes with reliable gene expression signals, 687 genes were differentially expressed (false discovery rate (FDR) < 0.05) between hot-evolved and reconstituted ancestral populations. In contrast, the cold-evolved control populations were very similar to the ancestral ones, with only 60 genes differing significantly. Because 35 of these genes were also differentially expressed between ancestral and hot-evolved populations, we attributed them to non-temperature-specific adaptation. These genes are enriched for oxidoreductase activity (eight cytochrome p450 genes, see Additional file 1: Table S1), suggesting a global down-regulation of detoxification genes.

Consistent with hot temperature adaptation affecting multiple genes, we identified a significant enrichment of several Gene Ontology (GO) categories and Kyoto Encyclopedia of Genes and Genomes (KEGG) pathways contrasting hot-evolved with ancestral or with cold-evolved populations. Genes down-regulated in the hot-evolved populations were enriched for more GO categories than up-regulated ones (Additional file 2: Table S2). Up-regulated genes were mainly enriched for defense response (including the Toll signaling pathway). Other categories overrepresented in up-regulated genes were triglyceride metabolism and cellular lipid metabolic processes, which include several genes involved in fatty acid synthesis or elongation (see Additional file 1: Figure S1). Down-regulated genes were enriched for a larger number of functions and pathways, most of which are related to metabolism: both the tricarboxylic acid (TCA) cycle and oxidative phosphorylation pathways were significantly down-regulated. Some key enzymes of glycolysis were also down-regulated (see Fig. 2a, b) along with the sucrose metabolism and carbon metabolism pathways.

### Resting metabolism measurements

With the transcriptomic data suggesting major regulatory changes affecting the metabolism, we reasoned that high-level metabolic phenotypes, such as respiration, should have changed as well. We quantified the resting metabolism by measuring CO_2_ emission overnight from the ancestral and both the cold- and hot-evolved populations. After 127 generations in the hot and 74 generations in the cold environment, we measured the CO_2_ emission of the evolved flies in parallel to a reconstituted ancestral population. In a generalized linear model (GLM) we identified the factors “population” (F_31,2_ = 5.7, *p* = 0.008) and “sex” (F_30,1_ = 13.7, *p* = 0.0008) to have a significant effect on CO_2_ emission (see Fig. 2e, none of the interactions between the factors was significant; *p* > 0.13). “Body weight” was not significant (F_30,1_ = 1, *p* = 0.3), because the difference in weight between sexes was already explained by the factor “sex” and females produced significantly more CO_2_ than males. We found similar results in a second series of measurements between the ancestral and hot-evolved populations after 133 generations (see Additional file 1: Supplementary Methods and Results).

### Genomic signature of adaptation

**Fig. 3:**
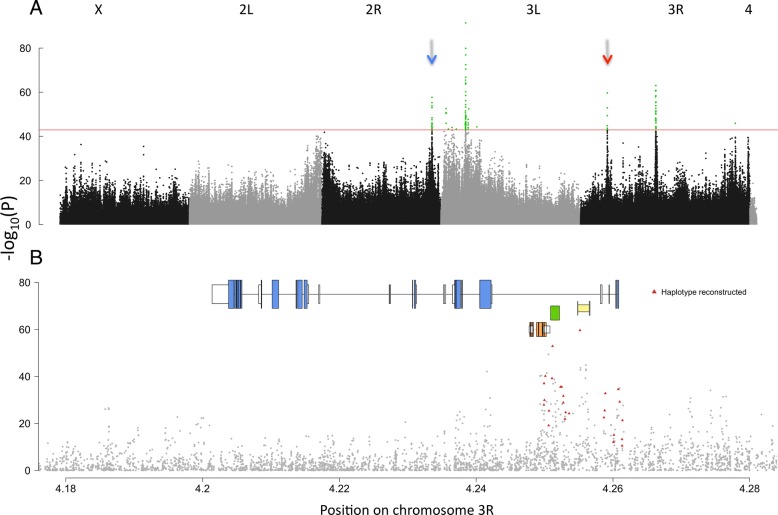
Genomic signature of adaptation to a hot laboratory environment. **a** Manhattan plot displaying the Cochran–Mantel–Haenszel *p* values of 2,741,793 SNPs (Methods). The *red line* indicates the *p* value cutoff for the most significant 100 SNPs (in *green*). The *red* and *blue arrows* indicate the *SNF4Aγ* and *Sestrin* peaks, respectively. **b** A close-up of Manhattan plot around the *SNF4Aγ* region. On top of the Manhattan plot the gene structure of *SNF4Aγ* is shown together with three small genes (*Snmp1*, *CG5810*, and *cDIP*; see Additional file 2: Table S3) located in one large intron of *SNF4Aγ*. Exons are indicated by *colored boxes* and introns by *thin lines*. *White boxes* indicate untranslated regions

While the transcriptomic response is well suited to identify pathways that are altered in response to temperature adaptation, it is inadequate for pinpointing the causal mutation(s) driving these changes. To map the targets of selection, we performed Pool-Seq [27] contrasts of the ancestral populations and hot-evolved flies at generation ~ 60 to identify genomic regions harboring pronounced allele frequency changes across all five replicates. Because the cold-evolved flies seem to be very similar to the ancestral flies for phenotypes that evolved in the hot environment, we only present here the genomic results of the hot-evolved flies. Based on the allele frequency changes of autosomal single-nucleotide polymorphisms (SNPs), we estimated an effective population size (*N*_e_) of 219 individuals (see Methods) in our populations. More than 2.7 million SNPs were tested for concordant allele frequency changes across replicates using the Cochran–Mantel–Haenszel (CMH) test. The Manhattan plot of the CMH –log10(*p* values) showed a handful of pronounced peak structures (Fig. 3a). Each peak comprises a set of linked SNPs that are highly differentiated between the hot-evolved and ancestral populations, a pattern indicating that the associated genomic regions likely carry selected variants. Using simulations to estimate a false positive rate, we retained the 100 most significant SNPs (false positive rate < 0.04), all contained in the five highest peaks of the Manhattan plot (Fig. 3b). While three peaks did not contain genes that can be directly linked to the observed changes in gene expression or their regulation (see Additional file 1: Supplementary Results), each of the two remaining peaks contained an interesting candidate gene, *Sestrin* and *SNF4Aγ*. Both genes are involved in metabolism homeostasis and interact with each other. The majority of the most significant SNPs in the peak of the 3R chromosome map to *SNF4Aγ* (although a large intron also contains three additional genes, see Fig. 3). The second peak of interest on the 3R chromosome arm was broader and included several genes, all with the same statistical support.

The highly pronounced peak structure in this experiment is different from the results of most evolve and resequence (E&R) studies in *Drosophila* (e.g., [28–34]). We think that several factors are probably responsible for this. First, the targets of selection start at rather high frequencies, which allows for recombination before the experimental evolution. Second, the use of *D. simulans* brings the advantage of no segregating chromosomal inversions and a higher recombination rate, in particular towards centromeres and telomeres [35–37]. Third, the use of a rather large number of founder chromosomes (about 1000) may also have contributed to a more pronounced peak structure.

### Characterization of the *Sestrin* and *SNF4Aγ* loci

While the mapping precision of the genomic region containing *Sestrin* is not very high (see Additional file 1: Supplementary Methods and Results), the selection signature for *SNF4Aγ* is narrow, encompassing less than 15 kb (Fig. 3b). We further refined the selection signature by looking for correlated allele frequency trajectories across replicates (see Methods) and identified 28 SNPs that may reside on similar haplotypes in the ancestral population (Fig. 3b). These 28 candidate SNPs start from a mean frequency of 44% in the ancestral population and rise as high as 96% (replicate 3) in ~ 60 generations (mean increase 42%, Additional file 1: Figure S2). We estimated an average selection coefficient of 0.07 across the 28 SNPs of interest in the *SNF4Aγ* locus (Additional file 1: Figure S6). No significant selection signature was detected in the *SNF4Aγ* region in the cold-evolved populations (data not shown).

Around the *Sestrin* locus, we found a higher number of SNPs (95) distributed over a broader genomic region (~ 60 kb) than for *SNF4Aγ* (See Additional file 1: Figure S7). The region that responded to selection contained multiple genes, *Sestrin* being at the left end of this region. Only the joint analysis of RNA-seq and genomic data allowed the identification of the putative target of selection. The alleles of the selected haplotypes start from a lower frequency than in the *SNF4Aγ* region (mean ~ 20%, see Additional file 1: Figure S8) and increase by ~ 40%. With an estimated selection coefficient of 0.06 (Additional file 1: Figure S9), the selection strength of *Sestrin* was similar to that of *SNF4Aγ*. The broader genomic region around *Sestrin* can be explained by the lower starting frequency of the selected haplotypes encompassing *Sestrin*.

We validated our Pool-Seq-based inference of selected haplotypes by sequencing 12 evolved haplotypes from two different replicates. For both loci the candidate SNPs cluster nicely and distinguish selected from non-selected chromosomes (Additional file 1: Figures S10 to S14). When considering all SNPs of the peaks, we identified pronounced differences between the two regions. The *Sestrin* locus is characterized by a single selected haplotype, with very few differences among the selected chromosomes. The *SNF4Aγ* region, however, carries multiple selected haplotypes that show some evidence for recombination, as expected for a high starting frequency. Interestingly, the non-selected chromosomes all carry the same haplotype at the *SNF4Aγ* region.

### Estimating the contribution of *SNF4Aγ* and *Sestrin* to phenotypic change

**Fig. 4:**
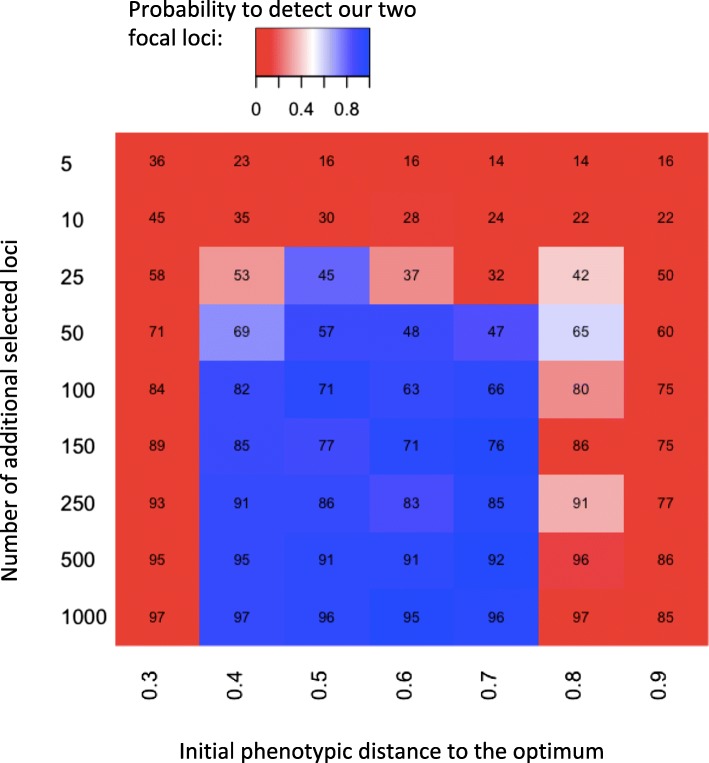
Two focal loci explain most of the phenotypic change, independent of the number of background loci. *Numbers* show the percentage of phenotypic change explained by the two focal loci, *color codes* the proportion of simulations in which they are significantly increasing in frequency. We used a quantitative trait model with a range of distances to the phenotypic optimum and different numbers of background loci contributing to the phenotype in addition to the two focal loci. With few additional loci of similar effect size (*first three rows*), the contribution of *SNF4Aγ* and *Sestrin* to the phenotypic change is minor, but the probability to detect them as outlier SNPs is low

The rapid frequency change of *SNF4Aγ* and *Sestrin* suggests that they are major-effect loci. Nevertheless, since the selected phenotype is not well defined, it is not possible to experimentally determine how much of the phenotype can be explained by these two loci. It is possible that, despite the pronounced frequency increase of *SNF4Aγ* and *Sestrin*, several loci of minor effect also contribute to the selected trait, such that only a small fraction of the phenotypic change can be attributed to *SNF4Aγ* and *Sestrin*. We evaluated this hypothesis using a quantitative genetics simulation framework [38]. We assumed that, in addition to *SNF4Aγ* and *Sestrin*, between 5 and 1000 loci contribute to the trait but were not detected in our study. The sum of the effect sizes of these background loci was set to be about ten times higher than the effect size of *SNF4Aγ* or *Sestrin*. Conditional on the observed rapid allele frequency change of *SNF4Aγ* and *Sestrin*, our simulations indicated that at least 45% of the phenotypic change could be explained by these two focal loci (see Fig. 4 and Additional file 1: Supplementary Results). Only when a small number of additional large-effect loci contribute to the trait do *SNF4Aγ* and *Sestrin* explain a smaller fraction of the phenotypic change. Nevertheless, in this case these loci also experience a frequency increase that would have been detected in our experiment (see Fig. 4). It is important to note that we assume that the other peaks in Fig. 3 which exceed the significance threshold do not contribute to the same trait as *SNF4Aγ* and *Sestrin*.

### *SNF4Aγ* variants in the North American and Australian latitudinal clines

Because the SNPs which responded to the new hot environment were present at intermediate frequency in the European population, we reasoned that they exhibit clinal variation in natural populations. Because the precise causative variant is not known for *SNF4Aγ* and *Sestrin*, it is not possible to test its distribution in natural populations directly. Nevertheless, we reasoned that the haplotypes carrying the target of selection may be partially preserved in other *D. simulans* populations; thus, we tested those SNPs of the selected haplotype block in our experiment that were also detected in clinal populations from two different continents [21, 23]. While for *Sestrin* no clinal signal was detected, out of 28 candidate SNPs in the *SNF4Aγ* region increasing in frequency in our experiment, 21 were shared with the US populations described by Machado et al. [21]. Eleven (52%) of these SNPs were at lower frequency in Maine than in Florida (see Additional file 1: Figure S14) and showed a clinal pattern among the Florida, Virginia, and Maine populations (Additional file 1: Figure S14). Samples from Pennsylvania, however, did not fit this clinal pattern and exhibited higher frequency than expected. From August to November, we observed a decrease in frequency of the hot alleles (i.e., those selected in the hot cages), an indication that our candidate SNPs could also be seasonal. Interestingly, despite the fact that the actual target of selection is not known, the clinal frequency change is highly significant (*p* < 0.008) based on Student’s *t* test for 10,000 random sets of SNPs generated by jackknifing 21 SNPs out of the 197 SNPs segregating in the region of interest (3R: 4,239,638-4,271,390, Additional file 1: Figure S15).

In the Sedghifar et al. [23] data set, we identified 15 and 23 SNPs from our list of 28 *SNF4Aγ* candidates in the North American and Australian populations, respectively. In the North American cline, we found a similar pattern as in the Machado et al. data set (see Additional file 1: Figure S16): in the Florida population, all the hot alleles were almost fixed (median allele frequency = 1), while they had lower frequencies in the Rhode Island population (median allele frequency = 0.94). This difference was significant based on the resampling test described above (see Additional file 1: Figure S16). In the Australian cline, the pattern was inversed, as the allele frequencies of our hot alleles were lower in the Queensland population (lower latitude, median = 0.77) than in the Tasmanian population (higher latitude, median = 0.87 (see Additional file 1: Figure S17). Interestingly, this trend is consistent with the observation of Sedghifar et al. that clinal SNPs were preferentially going in the opposite direction. In the light of this consistent trend, we conclude that the selected haplotypes in the Portugal population are also sufficiently conserved in Australia to generate a significant clinal signal.

## Discussion

### Novel thermal environment induces a rewiring of metabolic regulation

Temperature is a major factor modulating the expression of numerous genes in ectotherms and is particularly well studied in *Drosophila* [19, 20, 39]. Our experimental populations which evolved in a novel hot thermal environment displayed highly significant differences in gene expression involving many genes of well-defined pathways. Of particular interest were genes which were down-regulated in the hot-evolved populations, because they suggest a global down-regulation of energy production in hot-evolved flies, affecting glycolysis, TCA cycle, and oxidative phosphorylation pathways. Interestingly, a highly replicated study in *Escherichia coli* found that RNA polymerase was the most frequently targeted gene across replicates, resulting in a lower rate of protein synthesis [40], providing further evidence that an important evolutionary response to hot environments is to reduce the increase in energy production and protein synthesis, which is increased in hot environments and probably imposes a significant cost.

Consistent with modified metabolic rewiring of the hot-evolved populations, we found significant differences in CO_2_ production relative to the ancestral and cold-evolved control populations (see Fig. 2e; Additional file 1: Figure S3 and Supplementary Methods and Results). Contrary to naïve expectations, CO_2_ production was higher in the hot-evolved flies. Nevertheless, resting metabolism and gene expression are measured at two different moments of the daily cycle of the evolving populations, suggesting that the link between gene expression and energy production might not be straightforward. Additionally, higher CO_2_ production in hot-evolved flies is consistent with increasing O_2_ consumption associated with decreased AMPK activity [41]. Further insights into this counter-intuitive pattern of CO_2_ consumption come from a metabolomic analysis of *D. melanogaster* under a wide range of developmental temperatures [42]. At extreme temperatures the flies were depleted of sugars and energy metabolites (NAD+, NADP+, and AMP), which is attributed to their inability to maintain cellular homeostasis. If the hot conditions of our experiment have the same effect, flies not evolved to this environment may also be depleted of sugars and energy metabolites. In response, enzymes in the glycolysis, TCA cycle, and oxidative phosphorylation pathways could be up-regulated. Hot-evolved flies may have acquired the ability to maintain cellular homeostasis at high temperatures, allowing a higher resting metabolism without up-regulation of the metabolic pathway genes.

Our results contrast a recent study where CO_2_ production was conserved among *D. melanogaster* populations which evolved in different thermal environments [43]. With several experimental details differing between the studies (isofemale lines vs. pools of outbred individuals, 20 min measurements during the day vs. resting metabolism overnight), the interpretation of this apparent discrepancy is difficult. Nevertheless, it aligns well with the general controversy about the effect of temperature on the evolution of metabolism [44]. We conclude that the consistent differences in CO_2_ production between ancestral and evolved populations provide strong evidence of temperature-specific evolution of metabolism regulation but also indicate that the underlying physiological changes are more complex.

### AMPK explains the phenotypic changes observed in hot-evolved populations

Based on the genomic analyses alone, it is not possible to rule out other genes in the *Sestrin* peak as targets of selection, or three other small genes that overlap with the selection signature of *SNF4Aγ* (Additional file 2: Table S3). In combination with the expression data, however, the role of *SNF4Aγ* and *Sestrin* as the primary drivers of the metabolic rewiring becomes evident. *Sestrin* modulates the phosphorylation rate of AMP-activated protein kinase (AMPK) [45], which is composed of *SNF4Aγ* and two other subunits. AMPK is a key player in energy homeostasis at the cellular and the organismal levels, and both *SNF4Aγ* and *Sestrin* are directly linked to AMPK activity [45–47]. Low levels of ATP result in the activation of AMPK, which causes up-regulation of glycolysis and biogenesis of mitochondria [48]. Furthermore, energetically costly pathways, such as fatty acid production and gluconeogenesis, are down-regulated by AMPK [49]. Inactivation of AMPK causes down-regulation of glycolysis and up-regulation of anabolic pathways such as fatty acid production, which were both seen in our data. Interestingly, Pfk, the target enzyme for AMPK in glycolysis, is the first down-regulated enzyme of the glycolysis pathway in our data set (Fig. 2a, blue arrow). In *D. melanogaster*, RNA interference-mediated down-regulation of *SNF4Aγ* increases glucose content of muscles and the fat body [50] and induces starvation behavior [41]. Some of the genes of the insulin receptor signaling pathway were also differentially expressed in the hot-evolved populations (*Ilp6*, *InR*, see Additional file 1: Figure S1). Moreover, some key enzymes involved in fatty acid production (ACCoAs, ACC and FASN2, Desat1, CG30008, CG33110, CG18609; see Additional file 1: Figure S1) also show also signal of up-regulation, consistent with the direct inhibition of ACC by AMPK [51]. Increased temperatures and heat stress deplete fat storage in *D. melanogaster* [52] by invoking apoptosis in the fat body — a process dependent on *SNF4Aγ* [53] that links the starvation-like expression pattern observed here to temperature adaptation. *Sestrin* is also connected with autophagy regulation in *Drosophila*, through its role in activating AMPK [54, 55].

Thus, our results indicate that the activity of the key metabolic regulator AMPK is modulated through the differential regulation of the subunit *SNF4Aγ* and interacting gene *Sestrin* in hot-evolved populations. Given the central role of *SNF4Aγ* and *Sestrin* for temperature-dependent metabolic rewiring, we reasoned that both genes should vary along temperature clines in natural populations. While we did not find evidence for clinality of *Sestrin*, the patterns for *SNF4Aγ* matched our expectations. A whole genome polymorphism analysis identified *SNF4Aγ* as one of the top candidates in clinal North American *D. melanogaster* populations [22]. Clinal and seasonal variation of *SNF4Aγ* in *D. melanogaster* and *D. simulans* further implicate temperature as an adaptive driver [21, 24]. Reanalyzing clinal population genetic data [23], *SNF4Aγ* is among the 603 most differentiated genes shared by North American and Australian *D. simulans* populations. Gene expression of *SNF4Aγ* is clinal in European *D. subobscura* populations, with southern populations having lower expression levels [19], which parallels the response observed in our experimental evolution populations. Because the selected haplotype block may be partially maintained in other populations, we tested the diagnostic SNPs for clinal variation. Remarkably, populations from the extreme ends of the North American cline exhibit a clinal signal for the diagnostic SNPs. Nevertheless, the signal was mixed for less extreme populations.

### Large-effect loci segregating at intermediate allele frequencies drive rapid evolution

The combined analysis of transcriptomic and whole genome resequencing data of a freshly collected *D. simulans* population evolving in a new thermal environment identified two genes, both connected to AMPK, a central metabolic switch. While many possibilities exist as to how metabolism could be regulated, the strong selection response in all replicates suggests that two major-effect loci are driving the adaptive metabolic response in our populations. The observed selection signature clearly indicates that adaptation in our E&R study [27] is dominated by a small number of loci with strong effect, providing another example for rapid adaptation driven by a few major-effect loci [12–16].

The two haplotypes driving the metabolic switch in our experimental populations segregate at intermediate frequencies in the founder population and show clinal variation. Thus, it is highly plausible that these genes contribute to similar adaptive processes in natural populations, which probably occur over very short time scales. Because temperature varies seasonally, it is possible that spatial and temporal heterogeneity maintains the selected alleles at intermediate frequency in *D. simulans* [10, 24].

The fact that few large-effect loci resulted in a clear selection signature in our experiment does, however, not preclude that several minor effect loci also influence the metabolic rewiring in hot environments. Yet, our computer simulations suggest that these two loci probably explain more than 50% of the phenotypic change, even when minor effect loci are also contributing (Fig. 4). Previously, it had been shown that major-effect alleles contributing to quantitative traits show the fastest selection response, but with an increasing number of generations these loci are out-competed because small-effect alleles gradually increase in frequency [56]. The reason for the loss of the large-effect alleles is that it is easier to obtain genotypes close to the fitness optimum with small-effect alleles, while large-effect alleles could cause overshooting, resulting in more extreme phenotypes than favored by selection. Hence, the analysis of these experimental populations after a longer time interval could be very informative to understand the dynamics of adaptive alleles in natural populations.

With the favored allele being fixed or close to fixation in southern populations in the USA, it would be interesting to study the adaptive response in these populations. Because AMPK will probably not further contribute to adaptation, such an experiment could reveal other adaptive signals that were not detected in this study. Would such populations be segregating for other major alleles or would a polygenic response detected?

### Next steps

Experimental evolution provides an excellent framework for experimental testing of selected alleles. Allelic replacements with the CRISPR/Cas9 technology enable the direct comparison of selected and non-selected alleles in an otherwise homogeneous genetic background. Nevertheless, the mapping resolution in our study is still rather low. Replacing a genomic region of > 10 kb in *D. simulans*, a species with lower transformation efficiency than *D. melanogaster*, is extremely challenging. Thus, the next steps would require some further fine mapping of the target of selection. We anticipate that adding chromosomes without the selected alleles during an extended experimental evolution will provide more opportunity in recombination to obtain a smaller candidate region. Once sufficiently small candidate regions are cloned, many follow-up experiments are conceivable, ranging from competition experiments of selected and non-selected alleles in an experimental evolution setting to detailed biochemical comparisons using metabolomics, transcriptomics, and proteomics.

## Conclusions

We demonstrate with empirical data acquired at several biological levels (genomic, transcriptomic, metabolism, fecundity) that rapid adaptation to novel thermal environment involves rapid evolution of metabolism regulation that can be explained by large-effect alleles rising in frequency. We suggest that these alleles are maintained at intermediate frequencies in natural populations by temporal and spatial variations. Finally, we propose that E&R studies using different founder populations are a very powerful approach to answer questions about the genetic architecture of rapid adaptation, which are difficult to infer from natural populations.

## Methods

### Experimental evolution and common garden experiments

A detailed description of the experimental procedures can be found in Additional file 1: Supplementary Methods and Results. Briefly, ten replicated populations were created from a wild population of *Drosophila simulans* (Portugal 2008) and distributed randomly to two different selective regimes: a hot treatment with 12 h at 18 °C (dark) and 12 h at 28 °C (light) and a cold treatment with 12 h at 10 °C (dark) and 12 h at 20 °C (light). The replicate populations were then propagated the same way with non-overlapping generations and a census population size of 1000. After 64 and 39 generations, respectively, in the hot and cold treatments, we assayed all ten populations together with five replicates of a reconstituted ancestral population at 23 °C in a common garden experiment. Males aged 3–5 days old were frozen in liquid nitrogen and stored at − 80 °C for RNA sequencing.

### Pool-Seq analysis

Genomic DNA for pooled sequencing of the ancestral and the hot-evolved flies from generation F59 was extracted from females only. For each evolved population, DNA was extracted from about 500 females. Since only two replicates of the founder females were frozen (1250 females each), we added one replicate from generation F2 as a substitute. Genomic DNA was extracted using either the DNeasy Blood and Tissue Kit (Qiagen, Hilden, Germany) (for the two ancestral replicates) or a high salt extraction protocol [57] including RNase A treatment for the evolved populations. Paired-end libraries were prepared with different protocols and sequenced on different Illumina platforms (see Additional file 1: Table S6 for details). When the coverage of the first runs was not sufficient, we resequenced the populations at a later stage with a HiSeq2500 in 2 × 120 bp runs which required a modified adapter configuration (see Additional file 1: Table S6). The reads of the ancestral and the generation F2 replicates were combined and then randomly split into five artificial data sets to serve as replicated ancestral populations that match the number of replicates in the hot-evolved populations. SNPs were called with PoPoolation2, keeping only sites with at least one read with the minor allele per sample and coverage between 5 and 500. We masked sites flanking indels (± 5 bp) and repeats using PoPoolation2 [58] and RepeatMasker (www.repeatmasker.org, file available on demand). While library preparation protocols do not affect allele frequency estimates based on Pool-Seq [59], insert size does [60]. Therefore, we mapped the trimmed reads (quality ≥20 and length ≥ 50 bp) to the reference genome [61] using three different mappers (Bowtie2 [62], bwa-mem [63], and Novoalign [64]). Using only the SNPs called with all three mappers and restricting the analysis of each SNP to the mapper resulting in the least significant comparison (CMH test) prevents false positives [60]. We filtered for proper pairs and mapping quality ≥20 and finally retained 2,741,793 SNPs. We used Wright-Fisher simulations to estimate allele frequency changes expected in the absence of selection. Five independent simulation runs were performed, matching the initial frequency distribution of our ancestral populations and the number of SNPs tested in the original data set. We then added sampling noise to mimic the Pool-Seq process (binomial sampling) and conducted CMH tests on these simulated data, similarly as for the empirical data set. This way, we obtained a null distribution of CMH-based *p* values under a null hypothesis. Given a certain *p* value threshold, the false positive rate was computed as the fraction of simulated (neutral) and empirical loci. All simulations, sampling noise additions, and CMH tests were performed with the R package poolSeq [65].

There were 27 genes that contained at least one candidate SNP (Additional file 2: Table S3). Since *SNF4Aγ* was a good candidate to explain the observed phenotypic changes, we focused the subsequent analysis on a 200-kb region on chromosome 3R (4,150,000:4,350,000, see Fig. 2b). Reasoning that candidate SNPs may be located on one or a few haplotypes only, we used a haplotype reconstruction method that relies on the identification of SNP markers showing a correlated response across replicates and time points [66]. Allele frequencies for this analysis were based on Novoalign. Using the software package haploReconstruct [66], we identified SNPs with a correlation of least 0.95. We found 28 diagnostic SNPs (minimum coverage 20, minimum allele frequency change 0.2 in all five replicates).

We estimated the effective population size (*N*_e_) using the method of Jonas et al. [67], which accounts for the sampling procedure of Pool-Seq (plan I) based on all polymorphic sites of chromosomes 2 and 3. We inferred the selection coefficient (*s*) based on an *N*_e_ estimate of 219 and mean allele frequency of the 28 SNPs across all three replicates at the start of the experiment and at generation 59 using the method of Taus et al. [65] (Additional file 1: Figure S6).

The same procedure was then repeated for the *Sestrin* locus (2R-17,520,000:17600000, see Additional file 1: Figure S7). Using the haplotype reconstruction method, we found 95 diagnostic SNPs (minimum coverage 20, minimum allele frequency change 0.2 in all five replicates) showing correlated allele frequency changes (Additional file 1: Figure S8). We estimated the selection coefficient (*s*) based on these 95 SNPs (Additional file 1: Figure S9) using the same *N*_e_ estimate (219).

Since the results of our experimental evolution study may be affected by inadvertent migration events, we also tested whether migration may have resulted in false positives, but we consider this unlikely (see Additional file 1: Supplementary Methods and Results).

### Haplotype sequencing and analysis

We crossed 24 males from two different evolved populations (replicates 1 and 3, F67) with a virgin female from our reference *D. simulans* strain M252 (Genbank BioSample SAMN02713493, [61]). DNA extraction and sequencing were then produced from a single F1 female from each of the 24 crosses. Genomic DNA was extracted using a high salt extraction protocol [57] including RNase A treatment, and paired-end libraries were prepared and sequenced on the Illumina HiSeq 2500 (see Additional file 1: Table S6 for details). Reads were trimmed and mapped using bwa-mem as described above for pool sequencing. Additionally, we only retained the 2,741,793 SNPs which were consistently called by all three mappers in the Pool-Seq analysis and analyzed in our CMH test (Fig. 3b). SNPs with a coverage lower than 10 for a given haplotype were considered missing. We generated Figures S10 to S13 of Additional file 1 using the function heatmap.2 from the package gplots in R. While it is likely that these sequences represent naturally occurring haplotypes, we cannot rule out that recombination during the isofemale line stage has created a haplotype that is unlikely to occur in the wild.

### Phenotyping

We contrasted the phenotypes of hot-evolved, cold-evolved, and ancestral populations in a common garden setting, where all populations were maintained for two generations in the target temperature of 23 °C. We note that hot-evolved and cold-evolved populations were separated from the founder population by a different number of generations because of the faster development at warm temperatures. This may imply that the differences between hot- and cold-evolved flies may reflect different generation numbers, rather than a different selection response. While it is not possible to compare flies from the two temperature regimes at the same generation, we note that CO_2_ production (see the subsequent discussion) was tested at generation 74 of the cold-evolved flies, which roughly matches the generation at which the expression profile of the hot-evolved flies was determined (F64). Because CO_2_ production suggests no metabolic difference between the ancestral and cold-evolved flies, we conclude that the observed gene expression differences related to metabolism are unlikely to reflect only different generation numbers.

#### Gene expression analysis

We performed gene expression analysis of flies evolved for 64 generations in the hot environment and 39 generations in the cold environment. For all 15 populations (five replicates each in the hot, cold, and ancestral population) we generated two RNA-seq libraries, each from different sets of males. Total RNA was extracted from 25 to 30 males using the Qiagen RNeasy Universal Plus Mini protocol (Qiagen, Hilden, Germany) with DNase I treatment according to the manufacturer’s instructions. Libraries were generated using the NEBNext® Ultra Directional RNA Library Prep Kit for Illumina (details in Additional file 1: Supplementary Methods and Results). Raw reads were trimmed using PoPoolation [68] (quality threshold 20, minimum length 40) and aligned to the *Drosophila simulans* reference genome using GSNAP ([69], version 2018-03-25 with the option –m 0.008) using a Hadoop cluster [70]. Throughout this study, we used the genome and annotation of Palmieri et al. 2015 [61] as a default reference. All statistical analyses were performed using R [71]. Read counts were determined with Rsubread [72], and differentially expressed genes were identified with edgeR [73, 74]. We normalized gene expression levels with the trimmed mean of M-values (TMM) method, restricting our analysis to the 70% most highly expressed genes (minimum mean count per million (CPM) 6.81, 9238 genes). We used negative binomial GLMs to estimate the effect of selection regime on gene expression. We then computed ad hoc contrasts to find differentially expressed genes between groups of interest. The Benjamini–Hochberg procedure was applied to control for false discovery rate (FDR < 0.05).

Using these differentially expressed genes, we performed KEGG pathway enrichments with the R package gage [75] using the logFC computed by edgeR. GO enrichment analyses were performed with GOrilla [76], where all genes retained after filtering were used as the background data set. We only considered genes differentially expressed in the comparison of the hot samples against the ancestral as well as against the cold populations. Furthermore, in the comparison of the ancestral against the cold population the |logFC| was smaller than 0.2 (reported in Additional file 2: Table S2).

#### Resting metabolism

Resting metabolism was determined by repeatedly measuring overnight CO_2_ emission using a stop-flow respirometry system (Sable Systems International, North Las Vegas, NV, USA). All replicates of evolved and ancestral populations were reared for two generations at 23 °C, controlling egg density (400 eggs per bottle). Flies were collected shortly after eclosion, and after 24 h males and females were separated and placed at low density in vials (25 flies/cm^3^) under CO_2_ anesthesia. After 48 h recovery, the CO_2_ emission of 3–5 day old males and females was measured. Each assay was conducted at 23 °C in the dark, overnight (at least 12 h). The flies from different replicates were randomly assigned to one out of eight chambers together with a small piece of fly food (2 cm^3^) to avoid starvation response and desiccation. Each chamber contained approximately 25–30 flies. During the assays, a multiplexer (RM8 Intelligent Multiplexer) was sequentially flushing the metabolic chambers. Each flushing cycle lasted 5 min at a constant flow rate of 50 μl/min. We obtained repeated measurements in 40-min intervals for each of the eight channels. After removal of water by passing through a magnesium perchlorate column, we measured CO_2_ in the flushed air with a CA-10A Carbon Dioxide Analyzer. For each flushing cycle we determined the total CO_2_ emission using the ExpeData software (Sable Systems International) with an in-house script (available on demand). At the end of each assay the flies were dried and weighed.

We conducted the assay after generation 127 (74) of the hot (cold) evolved populations contrasting all three populations (2 ancestral populations, 4 cold and 4 hot-evolved populations, 5 successive runs), and a second time after 133 generations (5 hot replicates and 4 ancestral replicates, 4 runs). We estimated the resting metabolism for each chamber as the average of the three lowest observations overnight [77]. During the first set of measurements (generation 127), one chamber was left empty as a negative control. Because the CO_2_ levels in the empty chamber were always very low compared to the CO_2_ levels of chambers containing flies, we used all eight chambers with flies for the second set of measurements (generation 133). We analyzed the data with linear models. The most complete model included fixed effects of sex, mean dried weight, population identity (ancestral, cold-evolved, or hot-evolved), and interactions of these explanatory variables. Model comparison and selection of fixed effects occurred by stepwise removal of non-significant interactions and main effects, using analysis of variance (ANOVA) *F* tests. The assumptions of the models (normality of the residuals and homogeneity of the variance) were validated by visual inspection of the residuals.

#### Fecundity assays

We conducted fecundity assays of the cold- (F78) and hot- (F133) evolved populations in parallel with a reconstituted ancestral population using the same common garden design as described above. The only modifications were that we performed two generations of density control rather than a single one and that the ancestral population was reconstituted from ~ 90–100 lines only. In parallel, we set up another common garden experiment that differed only in the temperature regime, which was not constant but cycled between 28/18 °C light/dark conditions.

Flies were collected shortly after eclosion and allowed to mate within a 24-h period. Approximately 60 flies were placed under CO_2_ anesthesia in separate bottles to estimate egg laying rates. We created two bottles for each replicate (30 bottles in total). Every day, all flies were transferred without CO_2_ anesthesia to fresh food, and the eggs laid were counted. We did not count the eggs laid during the first 24 h after anesthesia and recorded the next 5 consecutive days. After 6 days the flies were then sexed and counted. We determined the mean number of eggs per female in a 24-h interval for each replicated population and tested for differences between populations using linear models in R. Significance of the fixed effects was tested using ANOVA *F* tests and differences between populations using Tukey tests (using the multcomp library and appropriate contrasts). At 23 °C, we excluded the results of two hot-evolved replicates (the 3rd and 4th) due to problems with the density control, and the data from only three replicates were used.

#### Computer simulations

We used the framework of Franssen et al. [38] implemented in a Python script available on dryad (10.5061/dryad.403b2). It models allele frequency changes of selected loci, all contributing to the same quantitative trait. Each locus is defined by a relative effect size, and its initial frequency and all loci are independent. The model assumes that individual fitness is normally distributed along the phenotypic axis and allows varying the initial distance to the fitness optimum in the ancestral population. We assumed that our two focal loci, *SNF4Aγ* and *Sestrin*, contribute to the trait proportionally to their coefficient of selection and modeled additional loci with an equal effect size. Because the effect sizes of all selected alleles are parameterized relatively to each other (i.e., summing up to 100% of total potential effect size), we maintained the summed effect sizes of all alleles constant between simulations. Thus, the number of background loci is inversely proportional to their effect size. We randomly sampled their starting frequency in the distribution of initial allele frequency in our experimental population (polarized according to the reference allele). The model assumes a Gaussian fitness function. We fixed the standard deviation (0.2) as well as the minimum and maximum fitness values (0.5 and 1.5, respectively) of the function and allowed the mean fitness optimum to vary.

We conservatively assumed that all loci contributing to the adaptive phenotype in addition to *SNF4Aγ* and *Sestrin* (background loci) had about a four times larger effect size than *SNF4Aγ* and *Sestrin* together (summed effect size of background loci = 0.5, effect size of *SNF4Aγ* = 0.07, effect size of *Sestrin* = 0.06). We assume here that there could be ~ 8 additional loci of the same effect size of *SNF4Aγ* or *Sestrin* or ~ 80 loci with an effect size ten times smaller than that of *SNF4Aγ* or *Sestrin*.

We computed the initial mean phenotypic value of the population (0.18) assuming that the frequency distribution of the background loci matches the one of all SNPs (mean frequency: 14%). The maximal phenotypic value when both *SNF4Aγ* and *Sestrin* are fixed (0.32) is then only 15% of the potential phenotypic increase (the maximal value is 1). We performed computer simulations varying two parameters: the number of background loci (5 to 1000) and the phenotypic value that maximize fitness (0.3 to 0.7). For each parameter combination, we performed 50 independent simulations. For each simulation, we report the proportion of the observed phenotypic change explained by our two focal loci after 60 generations in five independent replicates. We used the allele frequency after one generation as the initial value to account for random variation between replicates. To compare these simulated results to our experiment, we also computed a CMH test. To match the experimental data for each simulated locus, we randomly sampled “reads” at F1 and F60 matching the coverage of a chosen SNP in the experimental data set. We then computed for each set of parameters the proportion of simulations in which at least one SNP was above our CMH cutoff. We matched the population size with the empirical estimate (*N*_e_ = 219).

### Reanalysis of latitudinal North American and Australian *D. simulans* populations

We analyzed two published data sets of *D. simulans* populations along a cline [21, 23]. First, we used *F*_ST_ to determine whether the *SNF4Aγ* and *Sestrin* regions were differentiated along the clines. Since *F*_ST_ values do not indicate the direction of allele frequency differences, we performed a second analysis testing specifically whether the difference in allele frequencies between northern and southern populations along a cline differs in the direction expected based on our experiment. Below, we describe the analysis for each of the two data sets separately.

We downloaded, trimmed, and mapped the raw data from Machado et al. [21] using the same pipeline as described above but using only a single mapper (Novoalign). All alignment files were converted into an mpileup file using SAMtools. We restricted our SNP-based analysis to the individual sequences that were collected from four populations from Florida, Virginia, Pennsylvania, and Maine (thus excluding the pools). The Pennsylvania population was sampled three times the same year in August, September, and November 2011. We called the 28 SNPs of *SNF4Aγ* occurring on a haplotype block in the Portugal population in each population. At all 21 positions, we classified each sample in four categories: homozygous for either allele, heterozygous, or unknown. As the coverage for each sample was relatively low (1.8-fold), all reads were used to determine the genotype of each individual. Based on this classification, we computed the frequency of all the rising *SNF4Aγ* variants along the cline for each position. Only SNPs were used for which we genotyped in at least half of the samples in each population, which reduced the number of SNPs to 20.

We downloaded, trimmed, and mapped the raw data from Sedghifar et al. [23] using the same pipeline as described above but using only a single mapper (Novoalign). All alignment files were converted into an mpileup file using SAMtools, and we computed *F*_ST_ between populations at each polymorphic position using PoPoolation2 [58]. We filtered for transposable element insertions and sites flanking indels (± 5 bp) and only retained SNPs with a minimum coverage of 10. The *F*_ST_ values were computed for all positions individually while accounting for different numbers of chromosomes in each pool (6,795,806 and 7,990,580 SNPs in the USA and Australian clines, respectively). Focusing on the SNPs with the strongest differentiation, we retained only the top 0.1% SNPs with the highest *F*_ST_ values, resulting in *F*_ST_ thresholds of 0.53 and 0.52 in the Australian and American populations, respectively. These SNPs mapped to 2053 and 1506 genes, out of which only 603 genes were shared. Additionally, we compared the allele frequencies of our SNPs of interest in the *SNF4Aγ* region across the two clines (*Sestrin* was not among the 603 shared genes). We called the SNPs on each cline separately and only retained SNPs with a minimum coverage of 30 in each of the sampled sites.

## Additional files

Additional file 1: Contains Supplementary Methods, Supplementary Results, **Tables S1**, **S6**, **S7** and **Figures S1**–**S17.** (PDF 4847 kb)

Additional file 2: Contains **Tables S2**–**S5.** in separated sheets. (XLSX 83 kb)

## References

[1] Barton NH, Keightley PD (2002). Understanding quantitative genetic variation. Nat Rev Genet.

[2] Le Corre V, Kremer A (2003). Genetic variability at neutral markers, quantitative trait loci and trait in a subdivided population under selection. Genetics.

[3] Pritchard JK, Pickrell JK, Coop G (2010). The genetics of human adaptation: hard sweeps, soft sweeps, and polygenic adaptation. Curr Biol.

[4] Rockman MV (2012). The QTN program and the alleles that matter for evolution: all that's gold does not glitter. Evol.

[5] Mackay TF, Stone EA, Ayroles JF (2009). The genetics of quantitative traits: challenges and prospects. Nat Rev Genet.

[6] Yang J, Benyamin B, McEvoy BP, Gordon S, Henders AK, Nyholt DR, Madden PA, Heath AC, Martin NG, Montgomery GW, Goddard ME, Visscher PM. Common SNPs explain a large proportion of the heritability for human height. Nat Genet. 2010;42:565. Available: https://www.nature.com/articles/ng.608. 10.1038/ng.608 PMC3232052 20562875

[7] Turner TL, Stewart AD, Fields AT, Rice WR, Tarone AM (2011). Population-based resequencing of experimentally evolved populations reveals the genetic basis of body size variation in *Drosophila melanogaster*. PLoS Genet.

[8] Franks SJ, Hoffmann AA (2012). Genetics of climate change adaptation. Annual Review of Genetics.

[9] Messer PW, Ellner SP, Hairston NG (2016). Can population genetics adapt to rapid evolution?. Trends Genet.

[10] Savolainen O, Lascoux M, Merilä J (2013). Ecological genomics of local adaptation. Nat Rev Genet.

[11] Lescak EA, Bassham SL, Catchen J, Gelmond O, Sherbick ML, von Hippel FA, Cresko WA (2015). Evolution of stickleback in 50 years on earthquake-uplifted islands. Proc Natl Acad Sci.

[12] vant Hof AE, Campagne P, Rigden DJ, Yung CJ, Lingley J, Quail MA, Hall N, Darby AC, Saccheri IJ (2016). The industrial melanism mutation in British peppered moths is a transposable element. Nature.

[13] Nachman MW, Hoekstra HE, D'Agostino SL (2003). The genetic basis of adaptive melanism in pocket mice. Proc Natl Acad Sci.

[14] Daborn PJ, Yen JL, Bogwitz MR, Le Goff G, Feil E, Jeffers S, Tijet N, Perry T, Heckel D, Batterham P (2002). A single P450 allele associated with insecticide resistance in *Drosophila*. Science.

[15] Bersaglieri T, Sabeti PC, Patterson N, Vanderploeg T, Schaffner SF, Drake JA, Rhodes M, Reich DE, Hirschhorn JN (2004). Genetic signatures of strong recent positive selection at the lactase gene. Am J Hum Genet.

[16] Pascoal S, Cezard T, Eik-Nes A, Gharbi K, Majewska J, Payne E, Ritchie M, Zuk M, Bailey N (2014). Rapid convergent evolution in wild crickets. Curr Biol.

[17] Scheffers BR, De Meester L, Bridge TC, Hoffmann AA, Pandolfi JM, Corlett RT, Butchart SH, Pearce-Kelly P, Kovacs KM, Dudgeon D (2016). The broad footprint of climate change from genes to biomes to people. Sci.

[18] Merilä J, Hoffman AA. Evolutionary impacts of climate change. In: Oxford Research Encyclopedia of Environmental Science. New York: Oxford University Press; 2016.

[19] Porcelli D, Westram AM, Pascual M, Gaston KJ, Butlin RK, Snook RR. Gene expression clines reveal local adaptation and associated trade-offs at a continental scale. Sci Rep. 2016;6 10.1038/srep32975. 10.1038/srep32975 PMC5013434 27599812

[20] Zhao L, Wit J, Svetec N, Begun DJ (2015). Parallel gene expression differences between low and high latitude populations of *Drosophila melanogaster* and *D. simulans*. PLoS Genet.

[21] Machado HE, Bergland AO, O'Brien KR, Behrman EL, Schmidt PS, Petrov DA (2016). Comparative population genomics of latitudinal variation in *Drosophila simulans* and *Drosophila melanogaster*. Mol Ecol.

[22] Fabian DK, Kapun M, Nolte V, Kofler R, Schmidt PS, Schlötterer C, Flatt T (2012). Genome-wide patterns of latitudinal differentiation among populations of *Drosophila melanogaster* from North America. Mol Ecol.

[23] Sedghifar A, Saelao P, Begun DJ (2016). Genomic patterns of geographic differentiation in *Drosophila simulans*. Genet.

[24] Bergland AO, Behrman EL, O'Brien KR, Schmidt PS, Petrov DA (2014). Genomic evidence of rapid and stable adaptive oscillations over seasonal time scales in *Drosophila*. PLoS Genet.

[25] Bergland AO, Tobler R, González J, Schmidt P, Petrov D (2016). Secondary contact and local adaptation contribute to genome-wide patterns of clinal variation in *Drosophila melanogaster*. Mol Ecol.

[26] Tobler R, Hermisson J, Schlötterer C (2015). Parallel trait adaptation across opposing thermal environments in experimental *Drosophila melanogaster* populations. Evol.

[27] Schlötterer C, Kofler R, Versace E, Tobler R, Franssen SU (2015). Combining experimental evolution with next-generation sequencing: a powerful tool to study adaptation from standing genetic variation. Heredity (Edinb).

[28] Orozco-terWengel P, Kapun M, Nolte V, Kofler R, Flatt T, Schlötterer C (2012). Adaptation of *Drosophila* to a novel laboratory environment reveals temporally heterogeneous trajectories of selected alleles. Mol Ecol.

[29] Turner TL, Miller PM, Cochrane VA (2013). Combining genome-wide methods to investigate the genetic complexity of courtship song variation in *Drosophila melanogaster*. Mol Biol Evol.

[30] Tobler R, Franssen SU, Kofler R, Orozco-Terwengel P, Nolte V, Hermisson J, Schlötterer C (2014). Massive habitat-specific genomic response in *D. melanogaster* populations during experimental evolution in hot and cold environments. Mol Biol Evol.

[31] Franssen SU, Nolte V, Tobler R, Schlötterer C (2015). Patterns of linkage disequilibrium and long range hitchhiking in evolving experimental *Drosophila melanogaster* populations. Mol Biol Evol.

[32] Jha AR, Miles CM, Lippert NR, Brown CD, White KP, Kreitman M (2015). Whole-genome resequencing of experimental populations reveals polygenic basis of egg-size variation in *Drosophila melanogaster*. Mol Biol Evol.

[33] Phillips MA, Long AD, Greenspan ZS, Greer LF, Burke MK, Villeponteau B, Matsagas KC, Rizza CL, Mueller LD, Rose MR (2016). Genome-wide analysis of long-term evolutionary domestication in *Drosophila melanogaster*. Sci Rep.

[34] Griffin PC, Hangartner SB, Fournier-Level A, Hoffmann AA (2017). Genomic trajectories to desiccation resistance: convergence and divergence among replicate selected *Drosophila* lines. Genet.

[35] Barghi N, Tobler R, Nolte V, Schlötterer C (2017). *Drosophila simulans*: a species with improved resolution in evolve and resequence studies. G3 (Bethesda).

[36] Baldwin-Brown JG, Long AD, Thornton KR (2014). The power to detect quantitative trait loci using resequenced, experimentally evolved populations of diploid, sexual organisms. Mol Biol Evol.

[37] Kofler R, Schlötterer C (2014). A guide for the design of evolve and resequencing studies. Mol Biol Evol.

[38] Franssen SU, Kofler R, Schlötterer C (2017). Uncovering the genetic signature of quantitative trait evolution with replicated time series data. Heredity.

[39] Chen J, Nolte V, Schlötterer C (2015). Temperature-related reaction norms of gene expression: regulatory architecture and functional implications. Mol Biol Evol.

[40] Tenaillon O, Rodríguez-Verdugo A, Gaut RL, McDonald P, Bennett AF, Long AD, Gaut BS (2012). The molecular diversity of adaptive convergence. Sci.

[41] Johnson EC, Kazgan N, Bretz CA, Forsberg LJ, Hector CE, Worthen RJ, Onyenwoke R, Brenman JE. Altered metabolism and persistent starvation behaviors caused by reduced AMPK function in *Drosophila*. PLoS One 2010;5:pii:e12799. 10.1371/journal.pone.0012799. 10.1371/journal.pone.0012799 PMC2942814 20862213

[42] Schou MF, Kristensen TN, Pedersen A, Karlsson BG, Loeschcke V, Malmendal A (2017). Metabolic and functional characterization of effects of developmental temperature in *Drosophila melanogaster*. Am J Phys Regul Integr Comp Phys.

[43] Alton LA, Condon C, White CR, Angilletta MJ (2017). Colder environments did not select for a faster metabolism during experimental evolution of *Drosophila melanogaster*. Evol.

[44] Messamah B, Kellermann V, Malte H, Loeschcke V, Overgaard J (2017). Metabolic cold adaptation contributes little to the interspecific variation in metabolic rates of 65 species of Drosophilidae. J Insect Physiol.

[45] Ho A, Cho CS, Namkoong S, Cho US, Lee JH (2016). Biochemical basis of Sestrin physiological activities. Trends Biochem Sci.

[46] Mihaylova MM, Shaw RJ (2011). The AMPK signalling pathway coordinates cell growth, autophagy and metabolism. Nat Cell Biol.

[47] Hardie DG (2007). AMP-activated/SNF1 protein kinases: conserved guardians of cellular energy. Nat Rev Mol Cell Biol.

[48] Reznick RM, Shulman GI (2006). The role of AMP-activated protein kinase in mitochondrial biogenesis. J Physiol.

[49] Burkewitz K, Zhang Y, Mair WB (2014). AMPK at the nexus of energetics and aging. Cell Metab.

[50] Ugrankar R, Berglund E, Akdemir F, Tran C, Kim MS, Noh J, Schneider R, Ebert B, Graff JM. *Drosophila* glucome screening identifies Ck1alpha as a regulator of mammalian glucose metabolism. Nat Commun. 2015;6:7102. 10.1038/ncomms8102. 10.1038/ncomms8102 PMC4455130 25994086

[51] Hutber CA, Hardie DG, Winder WW (1997). Electrical stimulation inactivates muscle acetyl-CoA carboxylase and increases AMP-activated protein kinase. Am J Physiol Endocrinol Metab.

[52] Klepsatel P, Gáliková M, Xu Y, Kühnlein RP: Thermal stress depletes energy reserves in *Drosophila*. Sci Rep 2016, 6. doi:10.1038/srep33667. 10.1038/srep33667 PMC5027548 27641694

[53] Lippai M, Csikós G, Maróy P, Lukácsovich T, Juhász G, Sass M. SNF4Aγ, the *Drosophila* AMPK γ subunit is required for regulation of developmental and stress-induced autophagy. Autophagy. 2008;4:476–86. 10.4161/auto.5719. 10.4161/auto.5719 18285699

[54] Lee JH, Budanov AV, Park EJ, Birse R, Kim TE, Perkins GA, Ocorr K, Ellisman MH, Bodmer R, Bier E (2010). Sestrin as a feedback inhibitor of TOR that prevents age-related pathologies. Science.

[55] Budanov AV, Karin M (2008). p53 target genes sestrin1 and sestrin2 connect genotoxic stress and mTOR signaling. Cell.

[56] Yeaman S (2015). Local adaptation by alleles of small effect. Am Nat.

[57] Miller SA, Dykes DD, Polesky H (1988). A simple salting out procedure for extracting DNA from human nucleated cells. Nucleic Acids Res.

[58] Kofler R, Pandey RV, Schlötterer C (2011). PoPoolation2: identifying differentiation between populations using sequencing of pooled DNA samples (Pool-Seq). Bioinformatics.

[59] Kofler R, Nolte V, Schlötterer C (2016). The impact of library preparation protocols on the consistency of allele frequency estimates in Pool-Seq data. Mol Ecol Resour.

[60] Kofler R, Langmüller AM, Nouhaud P, Otte KA, Schlötterer C. Suitability of different mapping algorithms for genome-wide polymorphism scans with Pool-Seq data. G3 (Bethesda). 2016; 10.1534/g3.116.034488. 10.1534/g3.116.034488 PMC5100849 27613752

[61] Palmieri N, Nolte V, Chen J, Schlötterer C (2015). Genome assembly and annotation of a *Drosophila simulans* strain from Madagascar. Mol Ecol Resour.

[62] Langmead B, Salzberg SL (2012). Fast gapped-read alignment with Bowtie 2. Nat Methods.

[63] Li H, Durbin R (2009). Fast and accurate short read alignment with Burrows–Wheeler transform. Bioinformatics.

[64] Novocraft. http://www.novocraft.com. Accessed December 2015.

[65] Taus T, Futschik A, Schlötterer C (2017). Quantifying selection with Pool-Seq time series data. Mol Biol Evol.

[66] Franssen SU, Barton NH, Schlötterer C (2017). Reconstruction of haplotype-blocks selected during experimental evolution. Mol Biol Evol.

[67] Jónás Á, Taus T, Kosiol C, Schlötterer C, Futschik A (2016). Estimating the effective population size from temporal allele frequency changes in experimental evolution. Genet.

[68] Kofler R, Orozco-terWengel P, De Maio N, Pandey RV, Nolte V, Futschik A, Kosiol C, Schlötterer C (2011). PoPoolation: a toolbox for population genetic analysis of next generation sequencing data from pooled individuals. PLoS One.

[69] Wu TD, Nacu S (2010). Fast and SNP-tolerant detection of complex variants and splicing in short reads. Bioinformatics.

[70] Pandey RV, Schlötterer C (2013). DistMap: a toolkit for distributed short read mapping on a Hadoop cluster. PLoS One.

[71] R Core Team (2014). R: A language and environment for statistical computing.

[72] Liao Y, Smyth GK, Shi W (2013). The Subread aligner: fast, accurate and scalable read mapping by seed-and-vote. Nucleic Acids Res.

[73] Robinson MD, DJ MC, Smyth GK (2010). edgeR: a Bioconductor package for differential expression analysis of digital gene expression data. Bioinformatics.

[74] Schurch NJ, Schofield P, Gierliński M, Cole C, Sherstnev A, Singh V, Wrobel N, Gharbi K, Simpson GG, Owen-Hughes T, Blaxter M, Barton GJ (2016). How many biological replicates are needed in an RNA-seq experiment and which differential expression tool should you use?. RNA.

[75] Luo W, Friedman MS, Shedden K, Hankenson KD, Woolf PJ (2009). GAGE: generally applicable gene set enrichment for pathway analysis. BMC bioinformatics.

[76] Eden E, Navon R, Steinfeld I, Lipson D, Yakhini Z (2009). GOrilla: a tool for discovery and visualization of enriched GO terms in ranked gene lists. BMC bioinformatics.

[77] Jensen P, Overgaard J, Loeschcke V, Schou MF, Malte H, Kristensen TN (2014). Inbreeding effects on standard metabolic rate investigated at cold, benign and hot temperatures in *Drosophila melanogaster*. J Insect Physiol.

[78] Mallard F, Nolte V, Tobler R, Kapun M, Schlötterer C. A simple genetic basis of adaptation to a novel thermal environment results in complex metabolic rewiring in *Drosophila*. European Nucleotide Archive:PRJEB27022. 10.1186/s13059-018-1503-4 PMC6100727 30122150

[79] Mallard F, Nolte V, Tobler R, Kapun M, Schlötterer C. A simple genetic basis of adaptation to a novel thermal environment results in complex metabolic rewiring in *Drosophila*. Dryad Digital Repository. 10.5061/dryad.936p0df 10.1186/s13059-018-1503-4 PMC6100727 30122150

