# Supplementary material for: A simple genetic basis of adaptation to a novel thermal environment results in complex metabolic rewiring in Drosophila
Source: Genome Biol. 2018 Aug 20;19:119. doi: 10.1186/s13059-018-1503-4 (PMC6100727; doi:10.1186/s13059-018-1503-4)
Supplement: Supplementary file 1 — Contains Supplementary Methods, Supplementary Results, Tables S1, S6, S7 and Figures S1–S17. (PDF 4847 kb) [file 13059_2018_1503_MOESM1_ESM.pdf]

Supplementary Information for

A simple genetic basis of adaptation to a novel thermal environment results in complex metabolic rewiring in Drosophila

François Mallard[1], Viola Nolte[1], Ray Tobler[1,2], Martin Kapun[1] and Christian Schlötterer[1,*]
correspondence to: christian.schloetterer@vetmeduni.ac.at

Supplementary Methods
Supplementary Results
Figures S1 to S17
Tables S1, S6, S7

Supplementary Methods

Drosophila simulans population sample

In summer 2008, we established 250 isofemale lines from a *Drosophila simulans* collection in Northern Portugal (Póvoa de Varzim). After about 10 generations in the laboratory, we generated 10 independent replicate ancestral populations. For each replicate we used five mated females from each of the 250 isofemale lines (1250 females in total). These females were distributed across five bottles containing 70 ml standard *Drosophila* medium.

Culture conditions during experimental evolution

The flies were propagated in two fluctuating temperature regimes: five replicates evolved in a hot treatment with 12 h at 18°C (dark) and 12 h at 28°C (light), the other five replicates in a cold treatment with 12 h at 10°C (dark) and 12 h at 20°C (light). The populations evolving in the two temperature treatments were processed in the same way, except that the time after which flies were transferred to a new bottle was adjusted to account for the slower development at lower temperatures. Approximately three days after eclosion of a new generation (four to five days in the cold treatment), all flies from the five bottles were combined. After careful mixing the adults within each replicate, five samples of 200 individuals each were transferred to fresh bottles. After 48h (72h) of egg-laying in the hot (cold) environment, adults were transferred again to fresh bottles for another 48h (72h), after which flies were either frozen or used for DNA extraction. To prevent selection for early fecundity, flies eclosing from the first transfer were only used if the second transfer did not

yield enough flies to maintain a population size of 1000 individuals per replicate (5 bottles with 200 flies).

Common garden experiments

Prior to phenotypic assays and RNA-Seq, all five replicate populations of the hot and the cold evolved treatment as well as from a reconstituted ancestral population [80] were maintained in a constant 23°C common garden environment for two generations to control for maternal effects.

At generation 64 in the hot and generation 39 in the cold environment the common garden experiments were set up from additional egg lays. These eggs were transferred to a constant temperature (23°C, 12:12h light/dark cycles). In parallel, an ancestral population was reconstituted from the isofemale lines as described in Tobler et al. 2015 [26], a procedure, which faithfully mirrors the allele frequencies in the founder populations [80]. During the common garden experiment and phenotyping, this ancestral population was maintained in parallel to the evolved ones. After one generation of acclimatization, groups of 300 eggs were transferred to fresh bottles (i.e.: density control). Shortly after eclosion, adults were collected and sexed under CO_2 anesthesia. Males were separated from females and recovered 24-36h from CO_2 treatment before being frozen in liquid nitrogen at 2pm (approx. 6h after the light cycle started in the incubators).

Gene expression analysis

The RNA was quality-controlled on agarose gels and quantified using the Qubit RNA HS or BR Assay kit (Invitrogen, Carlsbad, CA). We generated strand-specific barcoded mRNA

libraries using the NEBNext® Ultra Directional RNA Library Prep Kit for Illumina with a protocol modified to allow for a larger insert size than the default 200bp.

PolyA-mRNA was purified from 3μg total RNA and fragmented for 8 min. The 42°C incubation step in the first-strand synthesis and the 16°C step in the second-strand synthesis were extended to 30 and 90 min., respectively. Size selection for a target insert size of 330bp was performed using AMPure XP beads (Beckman Coulter, Carlsbad, CA). PCR enrichment was done using NEBNext Multiplex oligos following the recommended protocol with 12 PCR cycles and a 50 sec. extension step.

The final libraries were bead-purified, quantified with the Qubit DNA HS Assay kit (Invitrogen, Carlsbad, CA) and pooled in equimolar amounts. Samples from ancestral, cold and hot evolved replicates were combined in the same pool, i.e. sequenced in the same lane, to reduce batch effects. Libraries were sequenced using a single-read 50bp protocol on a HiSeq2500.

Two of these libraries had a too low coverage to be analyzed (Table S4) and we finally obtained data from 28 libraries (nine ancestral, nine cold and 10 hot evolved).

RNA-Seq quality control

We performed several analyses to test the quality of each library. First we determined coverage heterogeneity, i.e.: a 3′ bias, using the geneBody_coverage tool implemented in the RSeQC package [81]. Since the 3’ bias is most pronounced for long genes, we performed this analysis using the 20% longest genes of the *D. simulans* transcriptome (the bed file is available on demand). Strongly biased libraries were removed from all subsequent analyses (see Fig. S4 for an explanation of the cutoff used).

Based on 12 chorion and yolk protein genes we tested for female gene expression in male samples to identify female contamination due to sexing mistakes (Table S5). We excluded four outlier libraries that showed total log2 normalized expression of these genes higher than eight (see Fig. S5). After removing female contaminated libraries and libraries with 3´bias, a total of 20 libraries (five ancestral, seven cold and eight hot evolved libraries) remained for analysis. Spearman correlation coefficients between all libraries showed a correlation of at least 0.948 between libraries. Despite some heterogeneity among libraries, a multi-dimensional scaling plot did not show outliers. Therefore, we retained all 20 remaining samples for differential gene expression analysis.

Supplementary Results

Analysis of the peaks of genetic differentiation

The top 100 SNPs retained for the analysis were mainly clustered in 4 major peaks of allelic differentiation on chromosome arms 2L, 3L and 3R (see Fig3, Table S3). Further analysis using a clustering approach (restricted to the peaks containing *SNF4Aγ* and *Sestrin)* [38] showed that these peaks likely contain a single selected haplotype block. In addition to the two peaks described in the main text, we identified two additional peaks that must contain a selected variant. The first one encompasses a very large region of the 3L chromosome arm containing more than half of the top 100 SNPs (n=54) over almost 5Mb. It is thus difficult to identify potential targets of selection. Nevertheless, the highest significant SNPs are close to *Fie* that is involved in temperature sensing and none of the genes close to these most significant SNPs of this peak are involved in metabolism regulation. The last peak is located on the chromosome arm 3R and contains 24 of the 100 top SNPs. Most of them map to a single gene (*Ace*, 20 SNPs). This gene is known to be selected in natural populations for

insecticide resistance and we have evidence that the selection around this loci in our experiment is not temperature specific as it is also selected in our cold environment (unpublished data). Furthermore, we show that the selection response at *Ace* is driven by the cost of insecticide resistance in an insecticide free environment. Hence, we consider it unlikely that the observed changes at *Ace* are associated with metabolism regulation.

Analysis of low frequency ancestral alleles

In order to ensure that the high repeatability of the allele frequency changes observed across our 5 evolved populations was not due to accidental contamination during our experimental procedure, we looked for low frequency alleles in the ancestral populations increasing during our experiments. If some of the observed consistent signal of allele frequency increase is due to a combination of contamination and drift, then these replicate specific SNPs should be rare and not evenly distributed across the genome.

We called the SNPs that at very low frequency the ancestral population (<0.5%) but present at more than 30% frequency in one evolved population and absent in the remaining four evolved populations. Because we detected such SNPs in all replicates distributed along all major chromosome arms (see Table S7), we do not consider migration a strong evolutionary force in our experiments.

<u>Resting metabolism</u>

In a second series of measurements after 133 generations, neither the interaction body weight:population nor body weight alone were non-significant (P=0.68 and P=0.23 respectively). The difference between hot evolved flies and the reconstituted ancestral population was only significant if body weight was included as a fixed effect in our model (see Fig. S3), suggesting that in addition to sex, other factors affected body weight between

populations. Since body weight is an important factor influencing CO_2 emission, we included body weight as a random effect in a mixed linear model and found a significant difference between the two populations ($\chi^2_{3,4}$=0.98, P=0.045). Because this value is close to the 5% threshold, we additionally tested for significance using bootstrapping by generating 10,000 random data sets having the same distribution as in our null model and computed the test statistic for each of these data sets. The random data sets were created using the function 'simulate' from the package stats in R applied to our null model (a mixed linear model with no fixed effect and only containing the random effect). For each simulated data set, we then computed the mixed models with and without population as fixed effect. Finally, we calculated the log-likelihood ratio test statistic comparing these two models for each simulated data set and compared this distribution to the statistic obtained from the true data set. We found that simulated test statistics are higher than the true one only 4.3% of the time, confirming the significant differences between the hot evolved and reconstituted ancestral populations.

Computer simulations

For several parameter combinations the allele frequency changes (i.e. CMH p-values) did not match our experimental results (red background color). With few background loci (5,10, 25), the focal loci are not increasing sufficiently in frequency because their phenotypic effects are on the same order of magnitude as the background loci. For the remaining parameter combinations (blue), the two focal loci explained between 43 and 97% of the total phenotypic change of the population (see Figure 4). The simulations reliably reproduce our experimental results when the initial distance to the phenotypic optimum is larger than the summed contribution of our two focal loci (>0.3), but also less than twice this contribution. This is because when the ancestral distance to the phenotypic optimum is low enough, most of the phenotypic change is achieved by our two focal loci. Yet, as the ancestral distance to the

phenotypic optimum increases, there are more opportunities for low effect size loci to increase in frequency and the probability to detect them is increased.

Finally, we made the simplifying assumption that all background loci have the same effect size, which is unrealistic. Although we did not test this, we assumed that in the case of heterogeneous effect sizes of background loci, most of the unexplained phenotypic increase would be associated with the background loci with the strongest effect sizes.

References

80. Nouhaud P, Tobler R, Nolte V, Schlötterer C. Ancestral population reconstitution from isofemale lines as a tool for experimental evolution. Ecol Evol. 2016;6:7169–75.

81. Wang L, Wang S, Li W. RSeQC: quality control of RNA-seq experiments. Bioinformatics. 2012;28:2184–5.

Table S1

List of genes consistently down- or up-regulated in the contrast between the ancestral and the two evolved populations (laboratory adaptation). The table presents the log2 fold changes and p-values for each comparison ancestral-cold evolved and ancestral-hot evolved. Negative log2 fold changes indicate that the expression is decreased in the evolved populations (and reciprocally).

FBgn	Symbol	log2FC (Anc/Hot)	P (Anc/Hot)	log2FC (Anc/Cold)	P (Anc/Cold)
FBgn0034612	CG10505	-1.8212	9.20E-18	-1.0091	5.55E-06
FBgn0030332	CG9360	-1.5041	2.14E-17	-1.2137	6.14E-11
FBgn0032381	Mal-B1	-1.0457	6.76E-07	-1.2880	1.41E-08
FBgn0000473	Cyp6a2	-0.9832	2.74E-06	-1.2715	4.36E-08
FBgn0033065	Cyp6w1	-0.9489	1.60E-05	-1.3693	2.54E-08
FBgn0033204	CG2065	-0.9033	1.22E-10	-0.6662	1.38E-05
FBgn0033981	Cyp6a21	-0.7575	3.32E-10	-0.5281	2.95E-05
dsim_PG00121	-	-0.7506	6.14E-09	-0.7896	1.94E-08
FBgn0013772	Cyp6a8	-0.7065	4.76E-04	-0.7981	1.66E-04
FBgn0027600	obst-B	-0.6887	2.52E-08	-0.5110	7.34E-05
FBgn0013773	Cyp6a22	-0.6831	3.23E-05	-0.6628	1.13E-04
FBgn0000008	a	-0.6624	2.51E-08	-0.5290	2.23E-05
FBgn0014469	Cyp4e2	-0.5947	1.56E-06	-0.5199	7.50E-05
FBgn0038734	CG11453	-0.5904	1.85E-07	-0.5497	3.97E-06
FBgn0010053	Jheh1	-0.5764	3.39E-04	-0.6737	5.66E-05
FBgn0038194	Cyp6d5	-0.5691	9.14E-05	-0.5687	2.75E-04
FBgn0038516	P5cr-2	-0.4922	3.57E-05	-0.4878	5.41E-05
FBgn0030615	Cyp4s3	-0.4049	2.10E-05	-0.5020	3.24E-07
FBgn0261800	LanB1	0.2821	3.10E-03	0.3887	5.45E-05
FBgn0037000	ZnT77C	0.3725	5.99E-05	0.4084	1.95E-05
FBgn0259140	CG42255	0.4344	4.29E-04	0.5434	1.36E-05
FBgn0010241	Mdr50	0.4754	7.53E-07	0.3641	3.19E-04
FBgn0263973	jv	0.6652	8.96E-10	0.4187	1.57E-04
FBgn0265267	CG18258	0.7218	4.95E-11	0.4603	3.65E-05
FBgn0262880	CG43235	0.7389	6.85E-05	0.7188	1.86E-04
FBgn0029147	NtR	0.7559	2.62E-04	0.7701	2.45E-04
FBgn0032136	Apoltp	0.7580	2.34E-15	0.4370	9.15E-06
FBgn0041607	AsnS	0.7871	5.40E-06	0.6817	1.38E-04
FBgn0032322	CG16743	1.0781	4.99E-15	0.6308	6.81E-06
dsim_PG00244	-	1.1202	6.42E-05	1.6259	6.71E-09
FBgn0035619	CG10592	1.2504	2.14E-10	0.7589	1.11E-04
FBgn0051324	CG31324	1.4001	7.89E-21	0.6080	1.06E-04
FBgn0035620	CG5150	1.5397	2.30E-15	0.7918	3.42E-05
FBgn0033729	Cpr49Af	1.5669	9.66E-04	1.9010	1.02E-04
FBgn0039471	CG6295	1.7332	8.85E-10	1.2142	2.31E-05

Table S6.

Description of the extraction and library preparation methods used for each sample (genomic data).

Library	DNA isolation	Amount of starting material	Fragmentation method	Kit	Size selection method	Insert size (bp)	Polymerase	No. of PCR cycles	Sequencing platform	Read length (bp)
Founder females A	DNeasy Blood and Tissue Kit[1] including RNase A treatment	5 µg	Nebulizer	Illumina Paired-End DNA Sample Prep protocol[3]	Agarose gel	210	Phusion[4]	10	GAIIx	2 x 74
Founder females A	DNeasy Blood and Tissue Kit[1] including RNase A treatment	5 µg	Nebulizer	Illumina Paired-End DNA Sample Prep protocol[3]	Agarose gel	210	Phusion[4]	10	GAIIx	2 x 76
Founder females B	DNeasy Blood and Tissue Kit[1] including RNase A treatment	5 µg	Covaris S2[2]	Illumina Paired-End DNA Sample Prep protocol[3]	Agarose gel	340	Phusion[4]	10	HiSeq 2000	2 x 100
Founder females A & B	DNeasy Blood and Tissue Kit[1] including RNase A treatment	ca. 4 µg	Covaris S2[2]	NEBNext Mastermix Kit E6040L and NEB Multiplex Oligos (single-index) for Illumina[4]	AMPure XP beads[5]	380	NEB Q5[4]	6	HiSeq 2500	2 x 120
Hot F2	DNeasy Blood and Tissue Kit[1] including RNase A treatment	5 µg	Covaris S2[2]	Illumina[3] Paired-End DNA Sample Prep protocol	Agarose gel	340	Phusion[4]	10	HiSeq 2000	2 x 100
Hot F59 r1,r3,r5	High salt extraction protocol[43] including RNase A treatment	2 µg	Covaris S2[2]	Reagents of TruSeq Paired-End Sequencing Kit[3]; ligation of TruSeq single-index adapters followed by pooling of ligated fragments; PCR amplification of the multiplexed size-selected fragments using the TruSeq mastermix[3]	Agarose gel	270	TruSeq PCR Mastermix[3]	10	HiSeq 2000	2 x 100
Hot F59 r1,r3,r5	High salt extraction protocol including RNase A treatment	1 µg	Covaris S2[2]	Reagents of NEBNext Ultra DNA library preparation kit E7370L[4]; size selection of each individual library on agarose gels; PCR amplification of individual libraries using NEB Multiplex (single-index) primers[4] followed by pooling	Agarose gel	310	NEB Q5[4]	10	HiSeq 2000	2 x 100
Hot generation 59 replicate 2,4	High salt extraction protocol[43] including RNase A treatment	ca. 4 µg	Covaris S2[2]	NEBNext Mastermix Kit E6040L and NEB Multiplex Oligos (single-index) for Illumina[4]	AMPure XP beads[5]	380	NEB Q5[4]	6	HiSeq 2500	2 x 120
Haplotypes	High salt extraction protocol[43] including RNase A treatment	ca. 100 ng	Covaris S2[2]	NEBNext Ultra DNA II Library Prep Kit4	AMPure XP beads[5]	410	NEB Q5 Hot Start[4]	8	HiSeq 2500	2 x 125

1 Qiagen, Hilden, Germany
2 Covaris, Inc. Woburn, MA, USA
3 Illumina, San Diego, CA
4 New England Biolabs, Ipswich, MA
5 Beckman Coulter, Carlsbad, CA

Table S7.

Number of replicate specific SNPs detected in each evolved populations on the main chromosome arms.

	X	2L	2R	3L	3R	4
Hot - Rep 1	239	239	151	133	183	131
Hot - Rep 2	83	83	52	50	212	155
Hot - Rep 3	165	179	72	60	305	1
Hot - Rep 4	122	122	100	133	231	113
Hot - Rep 5	107	107	171	143	720	146

Fig. S1.

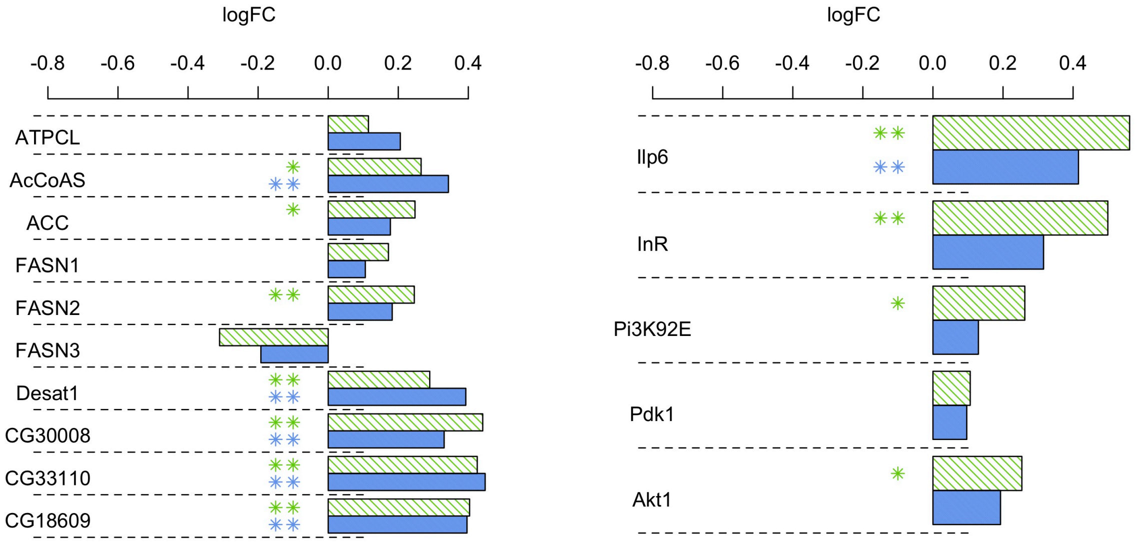

Barplots showing log2 fold change of expression between the hot evolved populations relative to the ancestral (green) or cold evolved (blue) populations. (** FDR<0.05, * FDR<0.1). Left panel: Fat metabolism genes. Right panel: Insulin signaling pathway genes.

Fig. S2.

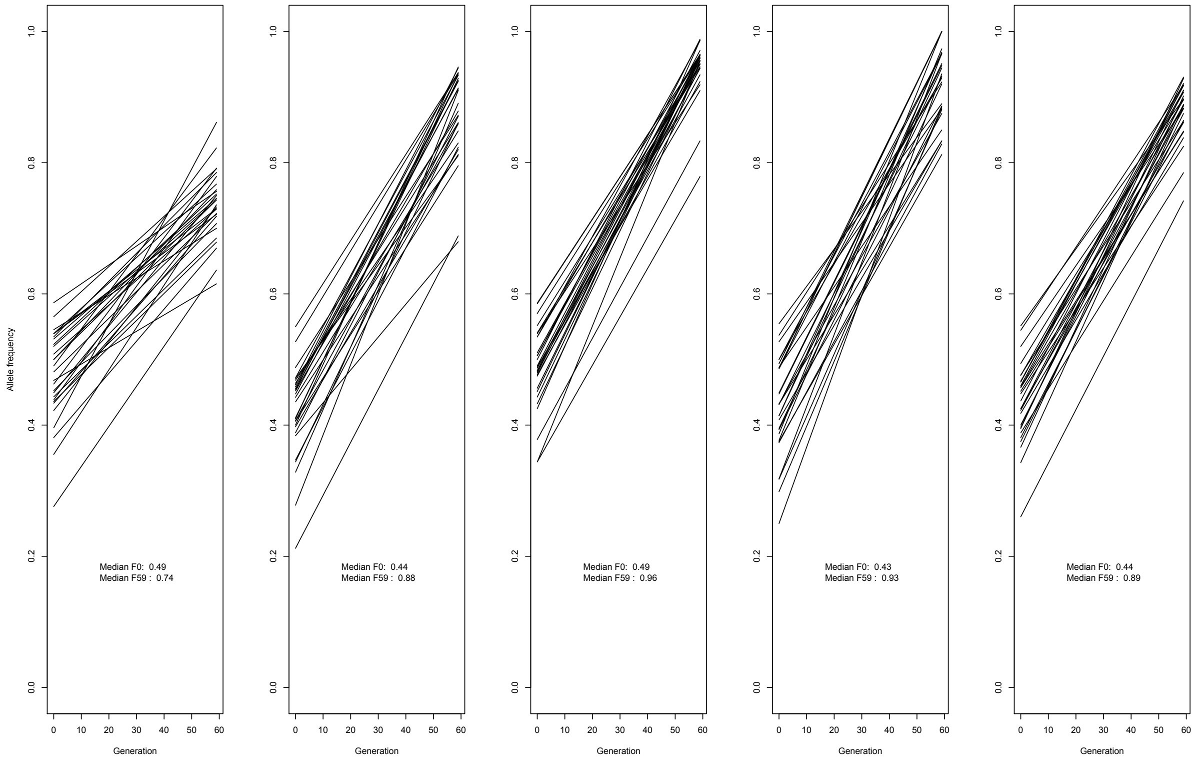

Allele frequency changes during 59 generations of experimental evolution for the 28 SNPs showing a correlated response across 5 hot evolved populations in the *SNF4Aγ* region.

Fig. S3.

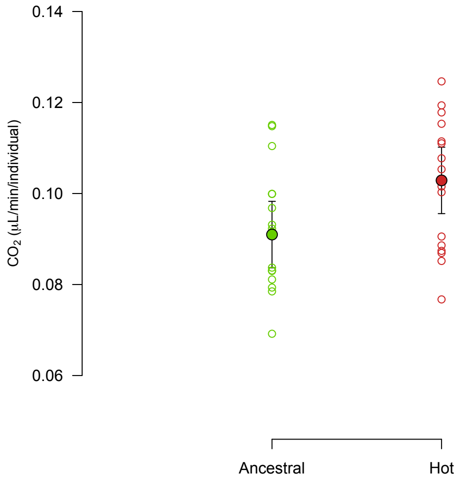

CO_2 emission is measured over two subsequent generations at 23°C. Although variable, the two mean emission is statistically different between the two populations when the variation in body weight between samples is accounted for. Closed symbols: mean with 95% confidence interval; open symbols: single measurements (n= 2*16), green: reconstituted base, red: population evolved in the hot environment for 133 generations.

Fig. S4.

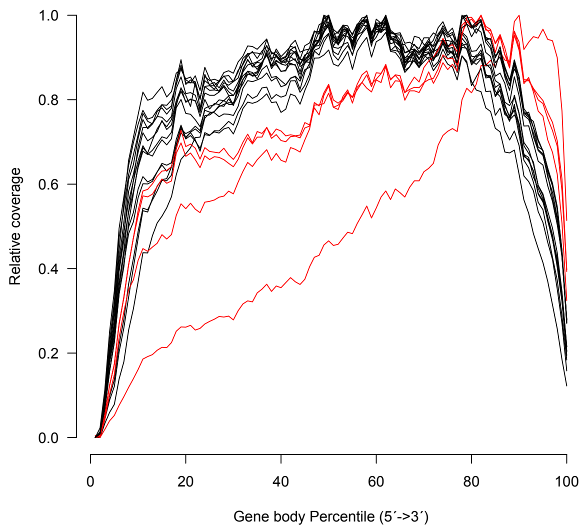

Relative coverage across genes for 28 RNA-Seq libraries. We removed three libraries (in red) from our analysis, because they exhibited a very pronounced 3' bias: at 50% gene body length the relative coverage was below 90%.

Fig. S5.

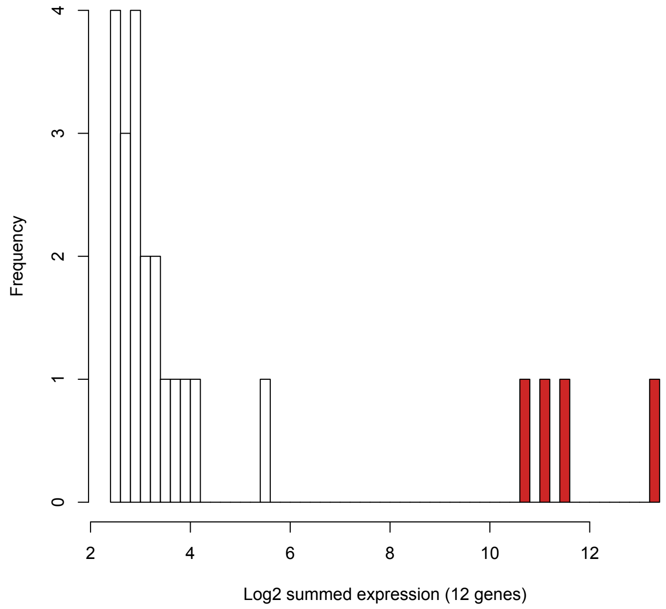

Identification of libraries with female contamination. We summed the expression of the nine chorion genes (CP15 to 19, CP36, CP38) and three yolk proteins (YP1 to 3). We excluded four outlier libraries (in red) with > 256 counts per million bp for the 12 indicator genes.

Fig. S6.

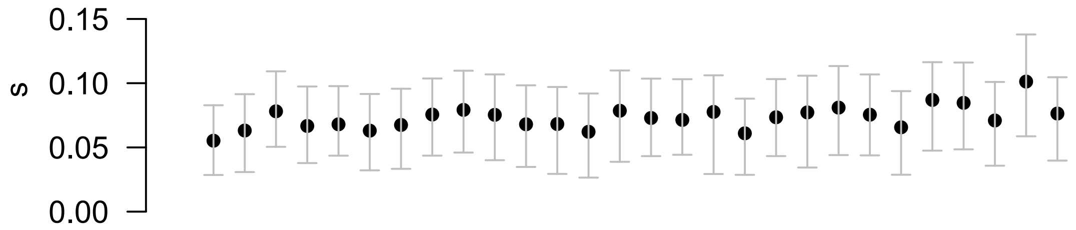

Estimation of the selection coefficient using the poolSeq R package individually for each of the detected SNPs in the *SNF4Aγ* region. Arrows represent 95% confidence intervals. All values are significantly different from zero ($p<0.01$). Median value = 0.07,

Fig. S7.

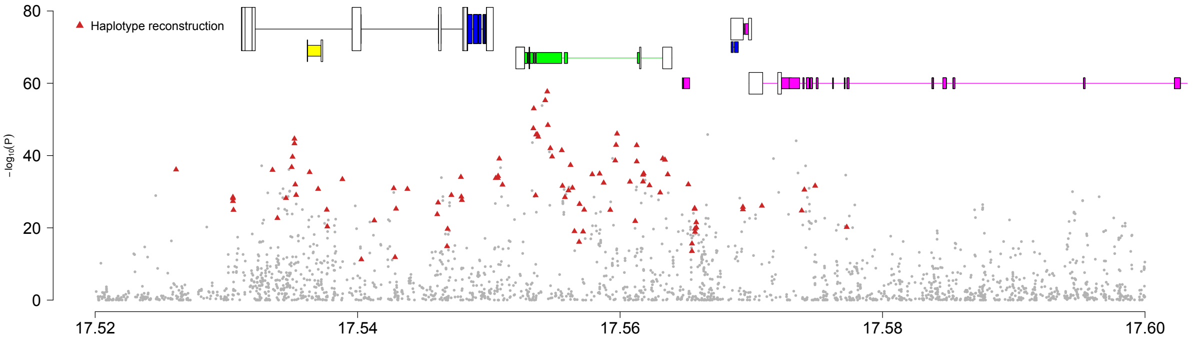

A close up of Manhattan plot around the *Sestrin* region. On top of the Manhattan plot the gene structure of *Sestrin* (leftmost blue gene) is shown. Exons are indicated by colored boxes and introns by thin lines. White boxes indicate UTRs. From left to right the genes are: *Sestrin* (blue), CG18128 (yellow), egl (green), CG13560 (purple), CG11300 (blue), CG5532 (small purple), CG9850 (long purple). Red triangles indicate 95 correlated SNPs, which characterize the selected haplotype(s).

Fig. S8.

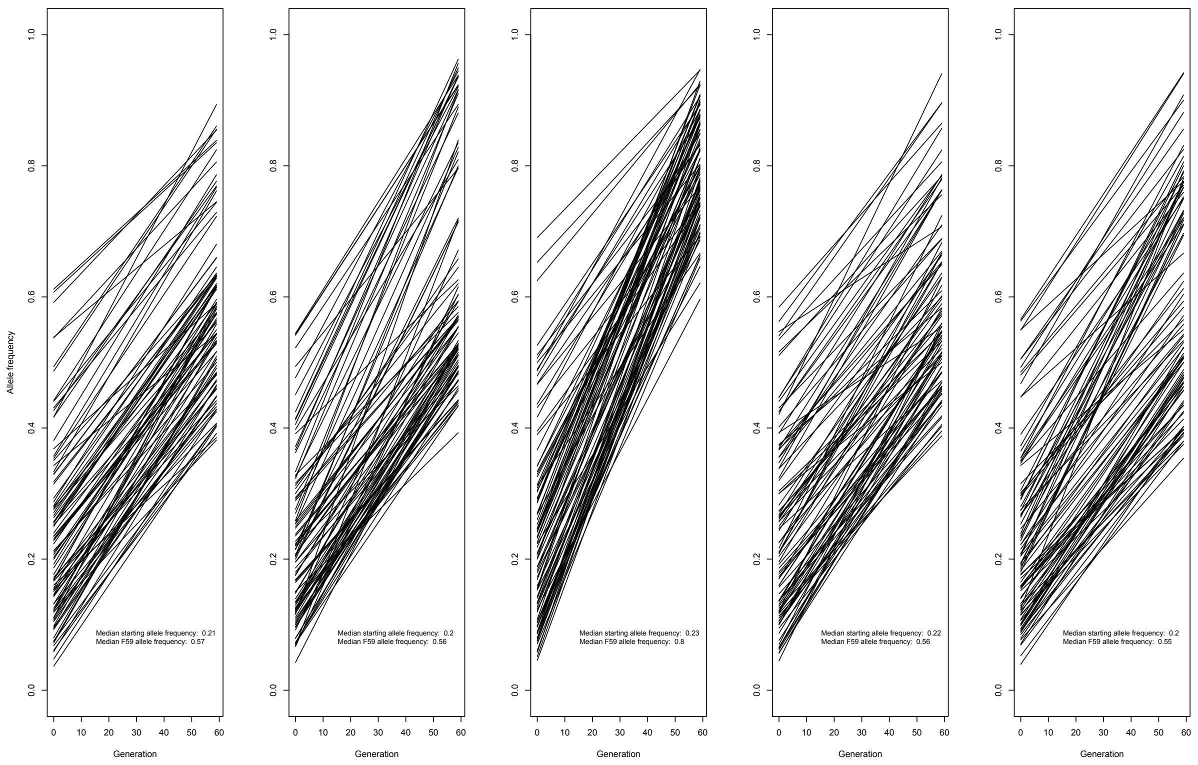

Allele frequency changes during 59 generations of experimental evolution for the 95 SNPs showing a correlated response across 5 hot evolved populations in the *Sestrin* region (2R: 17520000-17600000).

Fig. S9.

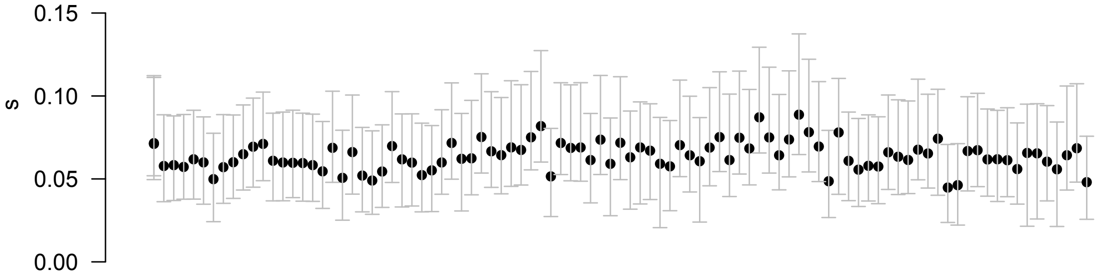

Estimation of the selection coefficient using the poolSeq R package individually for each of the detected SNPs in the *Sestrin* region. Arrows represent 95% confidence intervals. All values are significantly different from zero (p<0.05, 69 SNPs have p<0.01). Median value = 0.06,

Fig. S10.

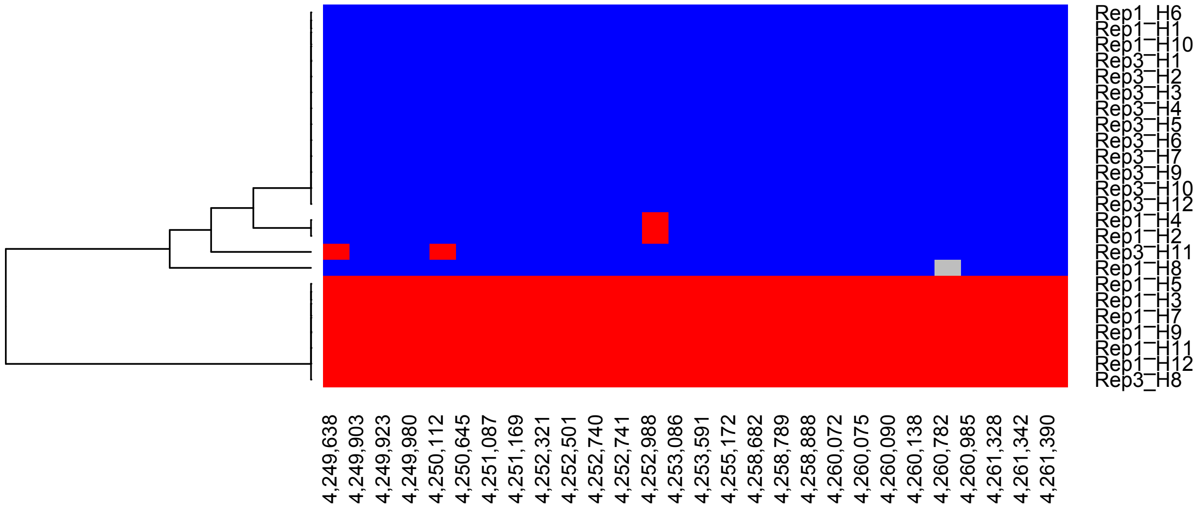

Haplotype structure in the *SNF4Aγ* region restricted to the 28 SNPs showing correlated frequency increase. We detected in both evolved populations (Rep1 and Rep3) some haplotypes carrying either all the selected alleles (in blue) or all the counter-selected alleles (in red). Missing values are plotted in gray.

Fig. S11.

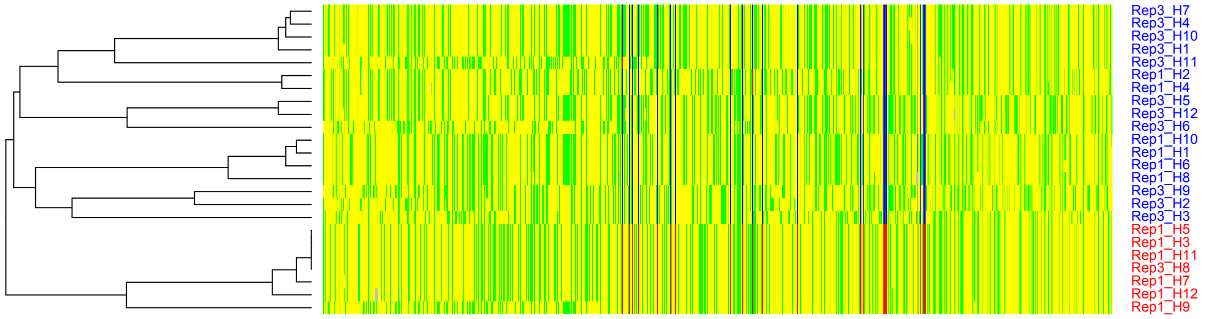

Haplotype structure in the *SNF4Aγ* region (10kb before and after the selected loci, 1106 SNPs, 3R:4,239,687:4,271,358). We detected multiple haplotypes containing all the selected alleles (blue labels), while the remaining ones show low variability. Yellow and green codes for non-selected polymorphism (ancestral and derived alleles respectively); missing values are plotted in gray color.

Fig. S12.

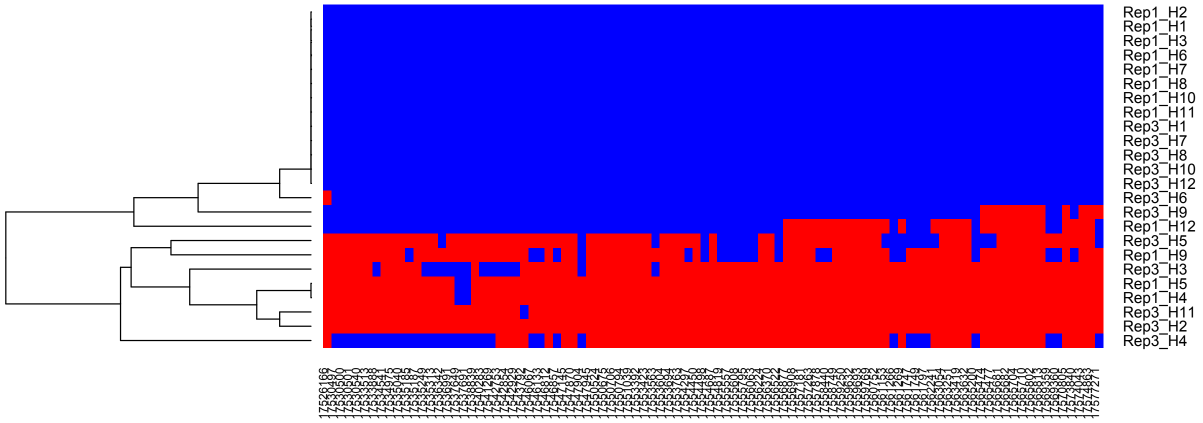

Haplotype structure in the *Sestrin* region restricted to the 95 SNPs showing correlated frequency increase. We detected in both evolved populations (Rep1 and Rep3) some haplotypes carrying either all the selected alleles (in blue). Missing values are plotted in gray and counter-selected alleles in red.

Fig. S13.

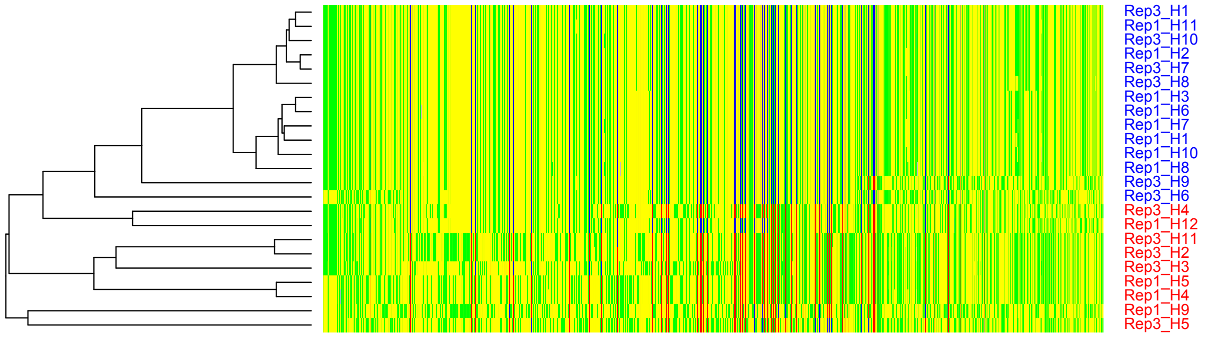

Haplotype structure in the *Sestrin* region (1444 SNPs, 3L:17,520080:17,587,103). We detected multiple haplotypes containing all the selected alleles (blue labels), while the remaining ones show low variability. Yellow and green codes for non-selected polymorphism (ancestral and derived alleles respectively); missing values are plotted in gray color.

Fig. S14.

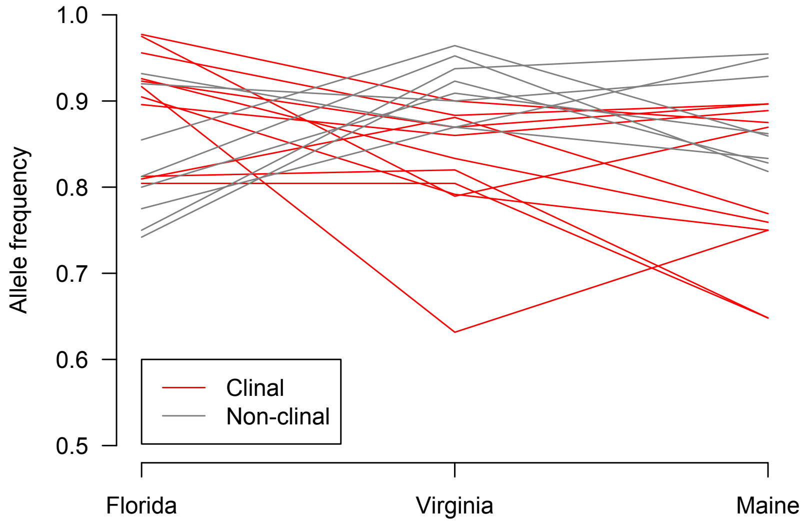

SNPs characterizing the selected haplotype at the *SNF4Aγ* locus in the experimental evolution study show clinal variation in natural populations (Machado et al.). 21 out of 28 SNPs had sufficient coverage in the Machado et al. data set. 11 SNPs displayed clinal variation. The decrease in frequency from Florida to Maine, is consistent with the observation that these alleles are favored in the hot experimental evolution cage.

Fig. S15.

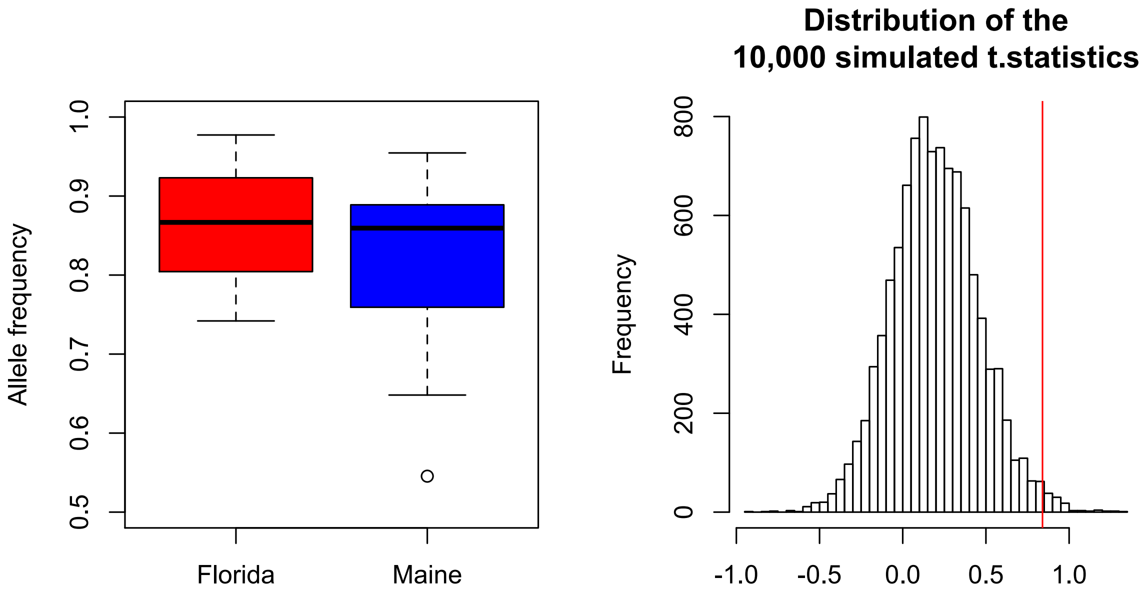

SNPs characterizing the selected haplotype at the *SNF4Aγ* locus in the experimental evolution study show clinal variation in natural populations: Machado et al.[5] data set. 21 out of the 28 SNPs had sufficient coverage in the Machado et al. data set. Left panel: Allele frequencies of the alleles favored in the hot cage in Florida (red) and Maine (blue). Right panel: Distribution of t.statistics obtained from random sampling of 21 SNPs in the region of interest. In more than 99% of the time the true set of SNPs (red vertical line) are more clinal than randomly sampled SNPs.

Fig. S16.

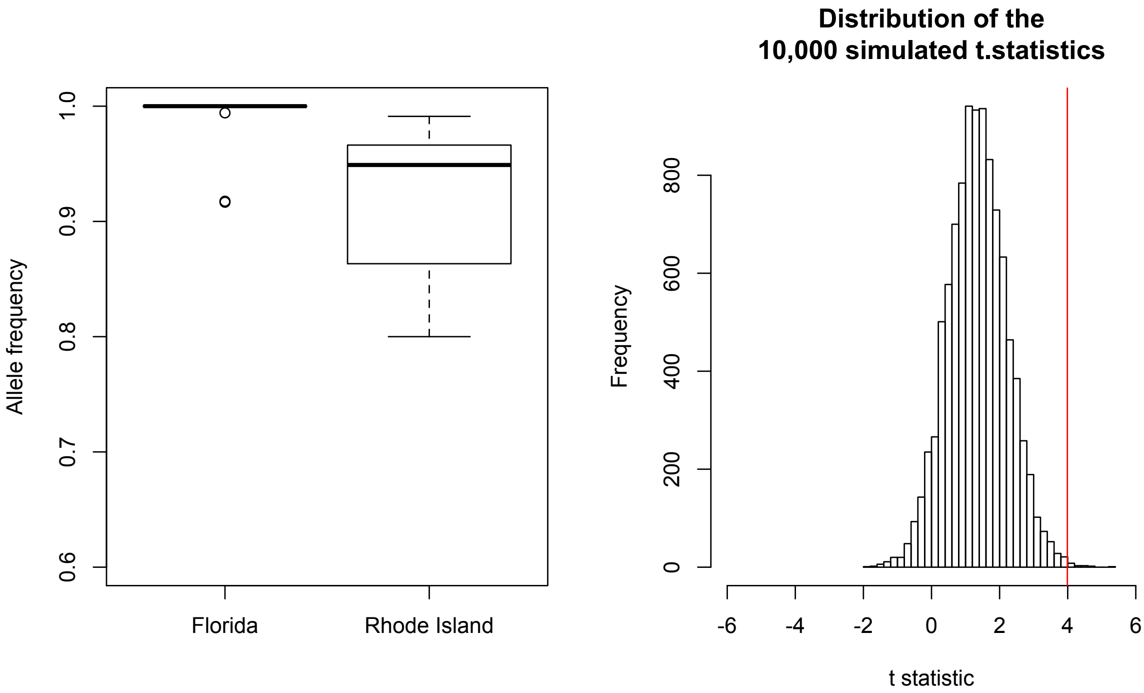

SNPs characterizing the selected haplotype at the *SNF4Aγ* locus in the experimental evolution study show clinal variation in natural populations: Sedghifar et al.[10] data set. 15 out of the 28 SNPs were also detected in the Sedghifar et al data set. Left panel: Alleles favored in the hot cage are found more frequently in the Florida (South) than in the Rhode Island (North, left panel, 15 SNPs). Right panel: Only 32 out of 10,000 of the t.statistics computed by random sampling of 18 SNPs from the 122 SNPs of the region of interest are lower than the one computed from the true set of SNPs. Left panel: Allele frequencies of the *SNF4Aγ* hot alleles found in the Sedghifar et al.[10]

Fig. S17.

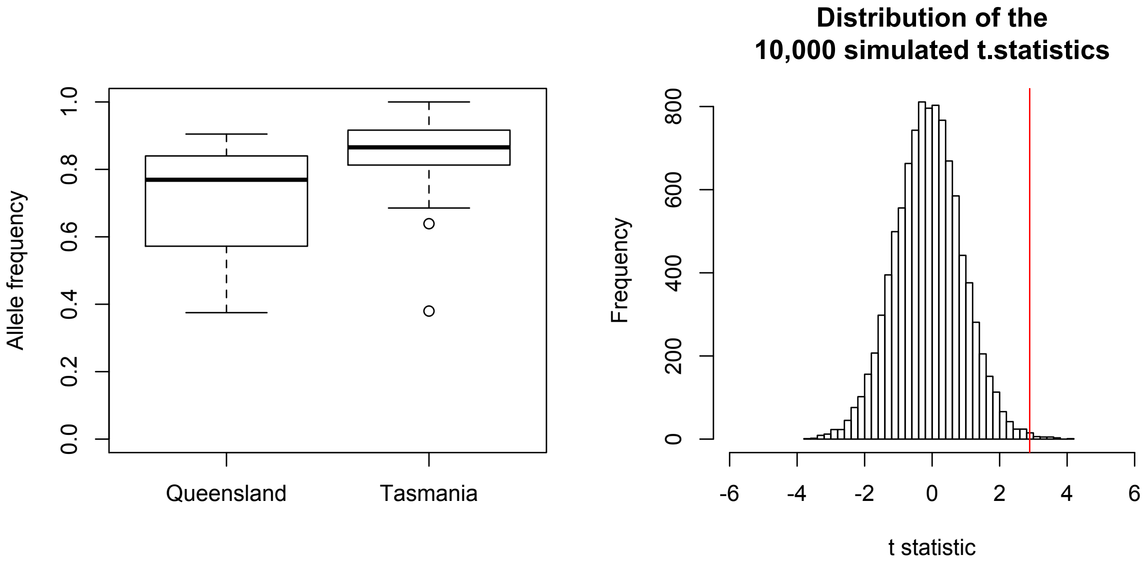

Australian populations. Here the clinal pattern is found in the opposite direction, hot alleles are less frequent in the lower latitude population (Queensland, median AF = 0.77) than in the higher latitude one (Ta smania, median AF=0.87, 23 SNPs). Left: Only 26 out of 10,000 of the t.statistics computed by random sampling of 25 SNPs from the 380 SNPs of the region of interest are lower than the one computed from the true set of SNPs.
